# Immune escape and metastasis mechanisms in melanoma: breaking down the dichotomy

**DOI:** 10.3389/fimmu.2024.1336023

**Published:** 2024-02-14

**Authors:** Carl A. Shirley, Gagan Chhabra, Deeba Amiri, Hao Chang, Nihal Ahmad

**Affiliations:** ^1^ Department of Dermatology, University of Wisconsin, Madison, WI, United States; ^2^ William S. Middleton Memorial Veterans Hospital, Madison, WI, United States

**Keywords:** immune escape, metastasis, EMT, hypoxia, acidosis, neural crest stem cell genes, dormancy, early dissemination

## Abstract

Melanoma is one of the most lethal neoplasms of the skin. Despite the revolutionary introduction of immune checkpoint inhibitors, metastatic spread, and recurrence remain critical problems in resistant cases. Melanoma employs a multitude of mechanisms to subvert the immune system and successfully metastasize to distant organs. Concerningly, recent research also shows that tumor cells can disseminate early during melanoma progression and enter dormant states, eventually leading to metastases at a future time. Immune escape and metastasis have previously been viewed as separate phenomena; however, accumulating evidence is breaking down this dichotomy. Recent research into the progressive mechanisms of melanoma provides evidence that dedifferentiation similar to classical epithelial to mesenchymal transition (EMT), genes involved in neural crest stem cell maintenance, and hypoxia/acidosis, are important factors simultaneously involved in immune escape and metastasis. The likeness between EMT and early dissemination, and differences, also become apparent in these contexts. Detailed knowledge of the mechanisms behind “dual drivers” simultaneously promoting metastatically inclined and immunosuppressive environments can yield novel strategies effective in disabling multiple facets of melanoma progression. Furthermore, understanding progression through these drivers may provide insight towards novel treatments capable of preventing recurrence arising from dormant dissemination or improving immunotherapy outcomes.

## 1 Introduction

Melanoma is a highly aggressive malignancy arising from melanocytes. Despite recent advances in melanoma research, the incidence of metastatic melanoma has only grown in the past decade (1–3). Treatments often fail to effectively overcome the adaptive mechanisms driving melanoma, especially in metastatic instances (4–11). Current immunotherapies for advanced stage melanomas involve antagonization of cytotoxic T-lymphocyte-associated protein-4 (CTLA-4, ipilimumab) and programmed cell death protein-1 (PD-1, nivolumab and pembrolizumab), but in 45-70% of cases these therapies encounter primary resistance (**Table 1**) (5, 11). Further, immunotherapies with initially promising responses eventually yield to cancer progression in ~20-30% of respondents as secondary resistance occurs (5, 13, 14). Moreover, relatively high recurrence rates persist among patients treated with immunotherapies and the 5-year survival rate for advanced melanoma rests around 40% (1, 9, 10). As melanoma progresses, the acquisition of an immunosuppressive milieu and alterations to endogenous pathways allow immune escape.

**Table 1:** Summary table of melanoma therapy standard of care recommendations from the American Society of Clinical Oncology 2023 guidelines (12).

Resection	Stage	BRAF Status	Therapy Type	Neoadjuvant Treatment	Primary or Adjuvant Treatment	Progression Status
YES	I-IIA	NA	NA	Not recommended	Not recommended	NA
	IIB/C		Immuno	Not recommended	1) pembrolizumab 2) nivolumab	
	III A/B/C/D	WT	Immuno	pembrolizumab for IIIB/C/D	1) pembrolizumab 2) nivolumab	
		Mutant (V600)	Immuno		1) pembrolizumab 2) nivolumab	
			Targeted		dabrafenib + trametinib	
	IV	NA	Immuno	pembrolizumab	pembrolizumab	
NO	III/IV	WT	Immuno	NA	1) nivolumab + ipilimumab followed by nivolumab 2) nivolumab + relatlimab 3) nivolumab 4) pembrolizumab	Progression on PD-1-based therapies? Switch to ipilimumab/ipilimumab-containing
		Mutant (V600)	Targeted		1) dabrafenib + trametinib 2) encorafenib + binimetinib 3) vemurafenib + cobimetinib	Progression on BRAF/MEK therapies? Switch to ipilimumab/ipilimumab-containing
			Immuno		1) nivolumab + ipilimumab followed by nivolumab 2) nivolumab + relatlimab 3) nivolumab	Progression on PD-1-based therapies? Switch to BRAF/MEK targeting

NA, Not available; WT, Wild Type.

The immune evasive capabilities of melanoma facilitate tumor survival, whereas its metastatic nature is what leads to lethality, as metastasis is estimated to cause about 90% of cancer deaths (15). Metastasis is a complex phenomenon involving sufficient lymphomagenesis or angiogenesis, intravasation, dissemination, resistance to anoikis and shear-stress induced apoptosis, extravasation, and subsequent resistance to a hostile metabolic and oxidative environment (15–20). Recent research has also challenged the linear progression model of cancer development, which suggests metastasis is a late-stage product of stepwise genetic and/or epigenetic changes in the primary tumor serving to gradually enhance cellular fitness and metastatic potential (21). Instead, the dissemination of metastasis-capable melanoma cells may be a common occurrence in the early stages (22–24). Malignantly juvenile cells escaping immunosurveillance may occupy a dormant phenotype for years before stimulation spurs metastatic colony formation and recurrence occurs (24). Immune escape and metastasis may initially appear as separate phenomena contributing individually to fatality, but they are intrinsically linked through individual drivers of melanoma progression. This becomes apparent in epithelial-mesenchymal transition (EMT), which has classically been viewed as the development of a stem cell-like, antiapoptotic, migratory, and progression-associated phenotype. Gathering evidence also associates EMT with the development of immune escape capabilities, and vice versa (25–30). “The chicken or the egg?” has been asked regarding the immunosuppressive environment and EMT (28). Immune escape and metastasis can be seen as two sides of the same coin—progression-associated mechanisms of melanoma development may simultaneously drive both migratory capabilities and subvert the immune response. Metastasis and immune escape are both critical factors in melanoma development that also inform therapeutic ventures and further research. This review focuses on discussing recently discovered drivers of melanoma progression that concurrently promote an immune evasive and metastatically inclined environment as well as future therapeutic directions.

## 2 Immune escape mechanisms in melanoma

Immune escape occurs when tumor cells avoid recognition and attack by the immune system. This phenomenon plays a key role in cancer progression and is linked to the effectiveness of immunotherapy (31). Malignant cells employ a variety of strategies to counteract antigenic recognition and the stimulation of the immune system. The escape of melanoma cells also relies on faulty immune recognition and increased resistance to apoptosis. An immunosuppressive microenvironment can also be vital in the escape of melanoma cells from the immune system (32). Below, we discuss important signaling mechanisms contributing to immune escape in melanoma.

### 2.1 Defective immune recognition

A major contributor to immune escape is defective immune recognition. Dysregulated antigen processing and recognition promote melanoma progression; inefficient antigen processing inhibits the ability of CD8^+^ T cells to recognize antigens on tumor cells (33). Antigens are displayed to CD8^+^ T cells through presentation as part of the major histocompatibility class-I complex (MHC I). It should be noted that the effectiveness of T cell cytotoxicity demands antigen presentation by mature dendritic cells (DCs). The co-stimulation and antigen presentation of DCs influence the melanoma immune response. During melanoma progression, immunosuppressant stimuli, such as interleukins (IL) IL-8 and IL-10, in the microenvironment can hinder DC maturation and lock DCs in immature phenotypes. This DC impairment is linked with decreased co-stimulation activity as a result of defective CD80 expression (34). Other populations such as myeloid-derived suppressor cells (MDSCs) and regulatory T cells (Tregs) accumulate in the melanoma milieu and contribute to imbalance between immune suppression and stimulation (35). Recruitment and active MDSCs can release soluble factors such as reactive oxygen species (ROS) including nitric oxide (NO), which can inhibit anti-melanoma abilities of natural killer (NK) cells and T-cells (36). Further, Tregs can inhibit the immune system through IL-10 and indoleamine 2,3-dioxygenase (IDO) overproduction. IL-10 and IDO similarly lessen the responses of NK cells, CD4^+^ and CD8^+^ lymphocytes against melanoma (37). Melanoma cells can also directly evade NK cell recognition by shedding MICA/B, which are recognized by the NK group 2D (NKG2D) receptor (38). Defective immune response is also linked to reduced levels of argininosuccinate synthetase in melanoma cells, leading to decreased production of arginine (39–41). When arginine is deprived, T cells show decreased survival rates and proliferation (42). Overall, multiple mechanisms and factors have been shown to be involve in defective immune recognition of tumor initiating cells, which influence the melanoma prognosis.

### 2.2 Immune checkpoint receptors

Immune checkpoints are a crucial control mechanism to inhibit T-cell responses and maintain self-tolerance. Dysregulation in immune checkpoint receptors represent another critical immune escape mechanism in melanoma. The most extensively studied immune checkpoint receptors are PD-1 and CTLA-4. PD-1 is a receptor that is involved in T-cell tolerance of self-antigens. Various studies have highlighted a ligand of PD-1, programmed death ligand-1 (PD-L1), as another factor in tumor immune escape. PD-L1 is expressed in non-blood cells such as astrocytes, neuronal cells, and keratinocytes (43, 44). PD-L1 demonstrates unusually high expression in tumor cells. The increased expression of PD-L1 by cells in the tumor microenvironment inhibits the function of cytotoxic T cells and apoptosis (45). Results from studies have shown an association between PD-1 expression on tumor infiltrating T cells and low chances of survival in cancer patients. In cutaneous melanoma metastases, PD-L1 has been shown to be frequently expressed (46). Indeed, the PD-1/PD-L1 pathway is the basis of many current immunotherapies, including melanoma (**Table 1**) (47).

The abnormal expression of immune checkpoint receptors and their binding capability by relative ligands has also been suggested as a mechanism to inhibit T cell activity in melanoma (48). This binding can initiate T cell exhaustion, diminishing T cell cytokine responses and cytotoxicity, and elevating expression of inhibitory surface receptors (49). These surface receptors include T-cell immunoglobulin mucin-3 (TIM-3), PD-1, Lymphocyte-Activation Gene 3 (LAG-3), CTLA-4, V-domain Ig suppressor of T cell activation (VISTA), and CD160 (50–54). Activation of these receptors also reduce IL-2 and Tumor Necrosis Factor-alpha (TNF-α) production (46). **Table 1** exemplifies the prevalence of immunotherapy in recommended melanoma treatment guidelines (12). Current investigation into connections between immune-related pathways and metastasis is an intense area of research that may lead to identification of new therapeutic targets and treatment options for melanoma management.

## 3 Immune escape and epithelial-to-mesenchymal transition

EMT is often considered a prerequisite to successful colonization of metastatic cells in distant organs and has been established as the critical mechanism responsible for acquisition of malignant phenotypes in epithelial cancers. During this transition, loss of epithelial polarity and adhesive factors are accompanied by simultaneous acquisition of a motile and stress-resistant mesenchymal phenotype (25, 55–57). Further, signaling associated with this process degrades the extracellular matrix to create an environment favoring dissemination (58). While melanocytes arise from the neural crest, they can still undergo dedifferentiation programs holding similar features to the classical EMT process. Melanoma “EMT” can be viewed as a phenotypic spectrum spanning between differentiated melanocytes and a dedifferentiated state (25). The concept of phenotype switching, which refers to the ability of cells or organisms to switch between multiple states/morphologies, is critical for melanocyte lineage differentiation, but also dedifferentiation into EMT-like phenotype transformations leading to metastasis (57). This process is associated with the increased expression of many immune checkpoints, as reviewed in (27), increased expression of mesenchymal markers or EMT transcription factors (EMT-TFs), and the simultaneous loss of epithelial markers (25, 57). Epithelial cadherins are replaced with N-cadherins and the expression of many EMF-TFs including Zinc finger proteins SNAI1/2, Zinc finger E-box-binding homeoboxes 1/2, and Twist-related protein (SNAI1/2, ZEB1/2, TWIST) are upregulated (25, 27, 55, 57). As melanoma progresses, EMT provides necessary intrinsic and extrinsic shifts promoting metastasis and post-disseminative survival (25, 58–60). These classical aspects of EMT are well realized, but an emerging paradigm is the two-fold contributions of EMT to both metastasis and immune escape. Below, we summarize recent literature showing connections between immune escape and EMT initiation, EMT-transcription factors and phenotype switching.

### 3.1 Immune escape and EMT initiation

Recent research suggests EMT is not a metastatically exclusive phenomenon as the dedifferentiated cell state is also associated with a variety of immunosuppressive properties. This first becomes apparent by examining the progressive framework behind EMT initiation. EMT-TFs can be initiated by modulations in a variety of pathways, including PI3K/AKT/mTOR, mitogen-activated protein kinase (MAPK), transforming growth factor-beta (TGF-β), wingless-related integration site (WNT), JAK/STAT, neurogenic locus notch homolog protein (NOTCH), SRY-Box Transcription Factor 10 (SOX10) among others (25, 57, 61–68). Activation of these pathways in melanoma simultaneously initiates EMT to promote metastatic and invasive phenotypes and deters the immune response. The multifaceted roles of important signaling cascades responsible for EMT directly tie metastasis to immune evasion. We have summarized the mechanisms of immune escape associated with these important signaling pathways leading to EMT and melanoma metastasis in **Table 2**.

**Table 2:** Immune escape impacts of commonly dysregulated EMT-inducing pathways.

Signaling pathway	Mechanisms of immune escape in melanoma	References
PI3K/AKT/mTOR	Immunosuppressants ↑ (IL-1, IL-6, IL-10, IL-35, TGF-β) Treg activity ↑ Context-dependent roles in B and T lymphocytes B cell development ↑ MDSC recruitment ↑ Suppressive monocyte recruitment ↑ “M2” Macrophage Polarization ↑ APC suppression ↑ PD-L1 expression ↑↓	(69–73)
BRAF/MAPK	Immunosuppressants ↑ (IL-1, IL-6, IL-8, IL-10, VEGF) Immunostimulants ↓ (IL-12, TNF-α) Antigen Presentation ↓ MHC Class I ↓ Lymphocyte infiltration ↓ MDSC recruitment ↑ Treg recruitment ↑ NK infiltration ↓ APC suppression ↑ STAT3 signaling ↑ PD-L1 expression ↑↓	(74–76)
TGF-β	Immunosuppressants ↑ (MCP-1, IL-8, IL-10, TGF-β, VEGF, CCL22, type 2 cytokines) Antigen Presentation ↓ MHC Class I and II ↓ CD40 expression ↓ Lymphocyte effectiveness and recruitment ↓ B cell infiltration ↓ MDSC recruitment ↑ Treg recruitment ↑ NK activation ↓ APC suppression ↑ Suppressive Dendritic Cell accumulation ↑ “M2” Macrophage Polarization ↑ Stimulation of MAPK, JAK/STAT, WNT ↑	(77–82)
WNT	Immunosuppressants ↑ (IL-6, VEGF, MMP-2, IL-8, IL-11, MCP-1) CD8^+^ T cell activity, infiltration, proliferation ↓ Treg accumulation and survival ↑ NK activation ↓ DC activity ↓ Enhanced MDSC suppression NF-κB activity ↑	(83–86)
JAK/STAT	Immunosuppressants ↑ (VEGF, MMP-2) MDSC recruitment ↑ Treg population ↑ CD8^+^ T cell killing ↓ NK tumor surveillance ↓ PD-L1 expression ↑ Interferon Resistance ↑ Immune stimulating cytokines ↓ HIF-1 ↑ p53 ↓ Macrophage crosstalk promoting WNT signaling ↑	(87–94)
SOX10	IRF1 ↓ CEACAM1 ↑ HVEM ↑ T cell activity ↓ PD-L1 expression ↑	(95–98)

↑: increased, ↓: decreased.

### 3.2 Immune escape and EMT-transcription factors

Common pathways leading to EMT only lay the foundation for EMT-related immune security. Increasing numbers of reports have suggested that EMT-TFs themselves play an active role in shaping the immune-regulation of melanoma. SNAI1 enhances production of C-C motif chemokine ligand (CCL) 2, which can subsequently induce EMT signaling in other cells of tumor microenvironment (TME) lacking endogenous SNAI1 while simultaneously contributing to the development of immunoregulatory DC populations. SNAI1-CCL2 signaling-induced regulatory DCs, which subsequently enhanced immunosuppressive Treg cells and impaired CD8^+^ T cells (99). SNAI1 overexpressing B16-F10 cells also displayed increased resistance to CD8^+^ T cell cytotoxicity in co-culture (100). SNAI1+ melanoma cells display enhanced production of TGF-β, thrombospondin-1 (TSP1), CCL2, and lipocalin 2 (LCN2), which may have additional immunosuppressive effects beyond Treg and DC regulation (99, 100). For example, CCL2 in TME has been shown to recruit MDSCs and macrophages (27).

Milk fat globule EGF-8 (MFG-E8) can be released by myeloid cells and was recently associated with melanoma progression and identified as an EMT trigger. In B16 cells, the migratory effects of MFG-E8 were diminished by TWIST knockdown. MFG-E8 also enhances Treg populations; this effect is mediated by TWIST activation in macrophages. Thus, TWIST is relevant as a target in both the microenvironment and melanoma-intrinsic signaling cascades (101).

Further, ZEB2 is essential for the development of terminal effector T cells, indicating modification of ZEB2 levels in T cells may be a mechanism promoting anti-immunity. When B16-F10 melanoma cells were co-cultured with T lymphocytes in hypoxic conditions, ZEB2 expression was inhibited with suppressed cytotoxic activity of T-cells showing a resistance strategy for immune escape (102). In human melanoma samples, high ZEB1 levels were found to be associated with decreased CD8^+^ T cell infiltration. A recent report utilizing a melanoma mouse model found ZEB1 expression decreases immunostimulant T-cell chemokines and impairs CD8^+^ T cell recruitment (103). ZEB1 has also been associated with M2 macrophage polarization and PD-L1 expression increases in lung adenocarcinoma, however, these factors remain to be explored in melanoma (103, 104). As the effects of EMT-TFs on immune escape are just emerging, additional research is required in the context of melanoma. Mechanisms behind EMT-TF driven immunogenicity in carcinomas are better documented and have been expanded to include effects on antigen presentation, MHC I expression, and immune checkpoint expression (25, 27). As the importance of EMT in metastasis and immunosuppression becomes apparent, investigation of other EMT-TFs and their interplay with the immune response is critical for understanding and controlling melanoma progression.

### 3.3 Immune escape and phenotype switching

EMT-TFs like SNAI1/2, ZEB1/2, and TWIST control the phenotype switching and are crucial for melanoma development. ZEB2/SNAI2 induces the expression of microphthalmia-associated transcription factor (MITF) and inhibits the expression of AXL receptor tyrosine kinase (AXL) to contribute to the MITF^high^/AXL^low^ phenotype. On the other hand, ZEB1/TWIST1 does the opposite and contributes to the MITF^low^/AXL^high^ phenotype (25). Evidence shows the ZEB2 to ZEB1 switch drives invasion and furthers the dedifferentiation of melanoma cells (25, 59, 60). High AXL resulting from EMT is associated with the upregulation of a myriad of genes governing both intrinsic immune evasion pathways and the release of immunosuppressants (29). Earlier studies in this area have shown how both high and low MITF populations evade the consequences of immunity through antigen, MHC, and immune checkpoint expression, inflammation, and deterring various lymphocyte activities (105–107). The relationship between EMT and immunity is further highlighted by looking at its relationship with immunotherapy. EMT is cited as a major obstacle for successful immunotherapy; the resulting phenotypes evade immunity through a plethora of intrinsic and environmental mechanisms (30, 108). In particular, the MITF^low^/AXL^high^ phenotype predicts resistance to a multitude of melanoma drugs (109).

Other markers beyond MITF and AXL can give insight to melanoma phenotypes produced by EMT. For example, increased nerve growth factor receptor (NGFR) can mark a post-EMT state (25) associated with T cell resistance (14) and NK cell resistance, enhancing metastatic potential (110). EMT is a process at the cellular level, but also the tumoral level. Various degrees of EMT-program induction and microenvironmental cues drive the formation of heterogeneous tumors, containing a wide range of phenotypic states with immune evasive and/or metastatic capabilities. Phenotype switching in the context of immune escape was recently reviewed in (111) and contributing to metastic progression in (112). Taken together, the signaling leading up to, during, and after an EMT-like dedifferentiation program enhances immune escape and metastatic ability at the cellular and tumoral levels.

## 4 Neural crest stem cell genes in EMT/metastasis and immune escape

Neural crest stem cells are a transient cell population in neural crest-derived tissues that arise during embryonic development and harbor stem cell properties. During embryonic development, melanocytes differentiate from neural-crest stem cells (NCSCs) (19, 113). Melanomas can manipulate genes important to NCSCs to achieve EMT-like characteristics involving growth, metastasis, and immune escape (113). In fact, neural crest (NC) cells experience EMT during normal development (114). The previously discussed transcription factors involved in EMT also play key roles in NC cell differentiation (25). Earlier review articles have discussed how NCSC programs and EMT are tightly linked (25, 114). Additionally, NOTCH, WNT, and SOX family members, as well as MITF, Paired Box 3 (PAX3), and Forkhead Box D3 (FOXD3) are essential for early neural crest development and have been reviewed in the context of initiating EMT in melanoma (57, 115–120). EMT reflects previous NCSC developmental programs and can hijack the same frameworks in melanoma progression. Below, we summarize recent reports of genes relevant to NCSCs being repurposed for immune escape and metastasis/EMT contributions in melanoma.

### 4.1 SHC adaptor protein 4

SHC4 is involved in the formation of epiblast stem cells (a pluripotent state that may give rise to neural crest stem cells) and later expressed in cells with neural identity (121). Normal melanocytes do not express SHC4, but its expression can be induced during the generation of malignant melanoma cells (19). A recent study in a melanoma mouse model found SHC4 reactivation greatly enhanced melanoma metastasis. In melanoma cell lines, ectopic SHC4 expression reduced cell adhesion, promoted amoeboid morphology of the tumor cells, and caused the activation of the MAPK pathway (19).

### 4.2 Brain-specific homeobox/POU domain protein 3A

BRN3A is not expressed in melanocytes, but is found to be expressed and required for cell cycle progression and survival in melanoma cells (122, 123). Histone Deacetylase 2 (HDAC2) promotes BRN3A expression and is a known attenuator of the immune response through regulation of PD-L1 expression, and its influences on antigen presentation (122). BRN3A also plays important roles in nervous system development. In melanocytic nevi, BRN3A cooperatives with RAS/RAF signaling to promote anchorage-dependent growth, which is a metastatic prerequisite (123, 124).

### 4.3 Special AT-rich binding protein-2

SATB2 is a transcription factor involved in neural development and migration. It regulates activation of many NCSC-related genes. Previous research has associated SATB2 expression with the metastatic MITF^low^/AXL^high^ and MITF^low^/NGFR1^high^/Aquaporin-1^high^ (AQP-1) genetic states (125). Another study has associated the MITF^low^/AXL^high^ state with immune escape through MHC I suppression and activation of TGF-β (126). A recent study further confirmed the ability of SATB2 overexpression to produce invadopodia and drive metastasis in a melanoma zebrafish model. Further, SATB2 was found to act as an EMT switch through activation of many EMT and NCSC programs (125).

### 4.4 Lysophosphatidic acid receptor 1

LPAR1 is present at high levels in NCSCs and certain melanoma cell lines but is either undetectable or barely expressed in melanocytes. In melanoma cell lines, its reactivation is shown to enhance migration and invasion (127). LPAR1 also activates yes-associated protein 1 (128), a known driver of immune escape and EMT in melanoma cell lines (82, 127, 129).

### 4.5 Neural cell adhesion molecule

NCAM plays an important role in controlling NCSC adhesion. NCAM expression drives malignancy in a multitude of cancer cell types. In A375 and M102 cell lines, NCAM knockdown reversed EMT marker expression and deterred migration (130). In addition, NCAM activates the AKT/mTOR pathway, its role as an initiator of EMT and contributor to immune evasion has already been discussed (69, 130, 131).

### 4.6 Bone morphogenetic proteins

BMPs are cytokines in the TGF-β family activated by growth differentiation factor 6 (GDF6), an important factor in neural crest development (132, 133). GDF6 developed a neural crest genetic fingerprint in A375 melanoma cell lines, and upregulated the NC markers SOX10, FOXD3, and SNAI2 while downregulating the growth inhibiting SOX9 protein. GDF6 was also linked to low levels of MITF, further demonstrating its ability to manipulate melanoma phenotypes (133). BMP signaling is present in 80% of melanomas but is absent in adult melanocytes. A recent study in a zebrafish melanoma model found BMP signaling has a large effect on early progression and contributes to the initiation of melanoma (134). The impact of BMPs on early progression, pathways controlling EMT, and their prevalence in melanoma specifically highlights the potent effects of NCSC genes. The power of certain NCSCs over metastasis and immune escape pathways combined with their silent nature in melanocytes provides an alluring avenue for future drug development.

### 4.7 Aquaporin-1

AQP-1 is an established NCSC marker (135). In a B16-F10 mouse model, an AQP-1 DNA vaccine was able to inhibit melanoma growth in a T cell dependent manner (136). The NCSC marker p75 neurotrophin receptor, also known as p75NTR/CD271/NGFR, acts as a lymphocyte suppressor through PD-L1 and antigen expression modulation (137). Past research has demonstrated AQP-1 overexpression in B16-F10 increases transwell migration (138).

**Figure 1:**
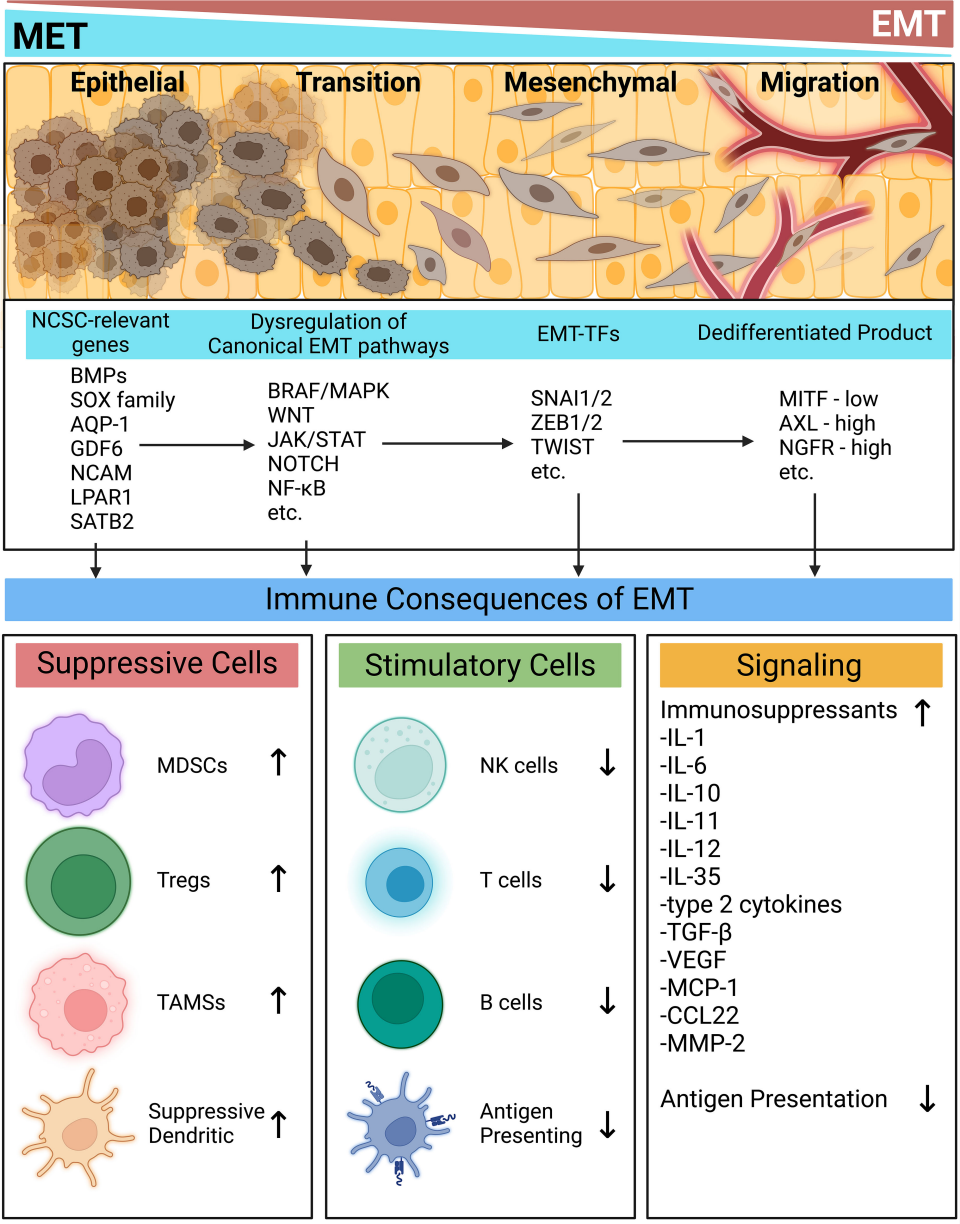
Immune implications of the epithelial-mesenchymal transition. At every stage of EMT, the activation of many signaling programs and their resulting phenotypes contribute to an immunosuppressive milieu. NCSC-related genes spur commonly dysregulated EMT-inducing pathways. EMT-TFs then act to modulate the melanoma phenotype. Each stage of this process contributes to intrinsic and extrinsic immune evasion mechanisms while simultaneously placing tumors in dedifferentiated states prone to metastasis. Created with BioRender.com. AQP-1, Aquaporin-1; AXL, AXL receptor tyrosine kinase; BMPs, bone morphogenetic proteins; BRAF, Serine/threonine-protein kinase B-Raf; CCL22, CC motif chemokine ligand 22; GDF6, growth differentiation factor 6; IL, interleukin; JAK, Janus kinase; LPAR1, Lysophosphatidic acid receptor 1; MAPK, mitogen-activated protein kinase; NF-kB, nuclear factor kappa B; NOTCH, neurogenic locus notch; MCP-1, monocyte chemoattractant protein 1; MDSC, myeloid derived suppressor cell; MITF, melanocyte inducing transcription factor; MMP-2, matrix metallopeptidase 2; NCAM, neural cell adhesion molecule; NCSC, neural crest stem cell; NGFR, nerve growth factor receptor; NK, natural killer; SATB2, special AT-rich binding protein-2; SNAI, snail family transcriptional repressor; SOX, SRY-related HMG-box genes; STAT, signal transducer and activator of transcription; TAM, tumor associated macrophage; Treg, regulatory T cell; TGF-β, transforming growth factor beta; TWIST, twist-related; VEGF, vascular endothelial growth factor; WNT, wingless and int; ZEB, zinc finger E-box binding homeobox.

Through EMT, melanoma acquires not only the ability to metastasize, but also to further fortify its immunosuppressive properties to the point of therapy resistance. The duality of metastasis and immune evasion can be seen throughout the EMT process. First in the pathways initiating EMT, the expression of EMT-TFs, and then in the properties and active genetic programs of the resulting dedifferentiated states. EMT is also tightly linked to NCSC-relevant genes, which may play a role in further fortifying the metastatic and immunoevasive properties of melanomas (**Figure 1**). EMT is crucial for melanoma progression and understanding the detailed mechanisms driving this process will result in development of novel therapies that simultaneously tackle immunity and invasion.

## 5 Immune escape in tumor hypoxia and acidification

Hypoxia, a prevalent feature of melanoma is characterized by a low oxygen level (<1%), compared to approximately 4–10% physiological oxygen concentration in different types of normal tissues and organs. Further, hypoxia is closely related to tumor acidification, a state of acidic tumor microenvironment (TME) (13, 40). Hypoxia and acidification are nearly unavoidable during development and progression of cancer, including melanoma (13, 40, 139–143). Initial tumor proliferation normally lacks accompanying vascularization; this quickly triggers oxygen depletion to create a hypoxic environment. Oxygen-sensitive pathways stimulated by hypoxia manifest themselves in a variety of metabolic genes. Anaerobic glycolysis becomes favored even in the presence of oxygen, leading to lactic acid accumulation in the TME (40, 143). The ability of hypoxia and acidosis to stimulate intrinsic melanoma pathways activating EMT, progression, and invasion is well studied (40, 139, 143–145). Hypoxia and acidosis are also able to reactivate NCSC genetic programs to bestow melanoma with stem-cell properties (40, 139). Oxygen-depletion also plays an important role in the tumor milieu, where it directly hinders T cell differentiation, drives T cell exhaustion, upregulates immune checkpoints and deters immunosurveillance (143, 144). Immune evasion is only further promoted by the acidic TME provided by glycolytic favoring. Acidic tumor pHs suppress lymphocytes including NK, innate, and T cells while simultaneously promoting tumor-associated macrophages (TAMs), Tregs, and MDSCs, and their immunosuppressive properties, through a multitude of mechanisms (143, 145). Acidosis also dysregulates intrinsic pathways building upon the effects of hypoxia-regulated ones, prevents anoikis, and promotes autophagy (141, 143).

An important recent study subjected melanoma tumors from 42 tumor infiltrating lymphocyte therapy-receiving patients and 74 immune checkpoint blockade-receiving patients to extensive proteomic analysis. In therapy responders, the authors identified crosstalk between mitochondrial activation pathways (including fatty acid oxidation, ketone body metabolism, the TCA cycle, and oxidative phosphorylation) and immune activation pathways (such as antigen presentation and type I and II interferon signaling). Fatty acid oxidation and ketone metabolism-related proteins promoted HLA expression *in vitro*, and their knockouts accordingly diminished T cell mediated cytotoxicity (146). Further, the high oxidative phosphorylation indicative of normal mitochondrial processes instead of hypoxic metabolism and glycolytic favoring was shown to be associated with positive immunotherapy outcomes. However, this study also suggested that the metabolic changes related to immune response were often associated with hypoxia (146). For instance, fatty acid oxidation can be increased by hypoxia (147), and to survive, hypoxic cancer cells could enhance fatty-acid metabolism and production (as reviewed in (148)). This contradiction may be explained by the presence of oxidative phosphorylation, which has been suggested to support the energy-intensive process of antigen presentation (146). The switch from oxidative phosphorylation to hypoxia could therefore increase energy demand from other metabolic pathways, replacing their potential pro-immune functions with pro-cancer ones. For example, it was previously shown that hypoxic conditions switch melanoma cells from using glucose to glutamine in the TCA cycle, which fuels proliferation (149). Carnitine palmitoyltransferase 1A (CPT1A) was identified as a key protein by the above discussed proteomic study (146), capable of enhancing T cell susceptibility and supporting antigen presentation. On the contrary, a recent study found that CPT1A-knockout B16 melanoma tumors were extremely susceptible with adoptive T cell therapy (150). However, differences in the oxidative phosphorylation/hypoxia status between these cases remains unknown. The relationship between hypoxia and other metabolic pathways, and how hypoxia can potentially reprogram these pathways to convert anti-tumor immune features into pro-survival assets, remains an open area of investigation that may further enhance the allure of targeting hypoxia.

Recent research also demonstrates that melanoma acidification driven EMT is reversible upon returning to normal conditions (139). The possibility to incapacitate, or even reverse, melanoma progression through any of these echelons makes hypoxia and acidosis alluring areas of study. Hypoxia and acidosis are prime examples of the duality between metastasis and immune escape. Below, we summarize recent advances into these phenomena and further highlight this congruency.

### 5.1 Recently revealed hypoxia and acidosis driving factors

#### 5.1.1 Ephrin type-B receptor-4

EPHB4 has been shown earlier to promote melanoma cell migration through Rho signaling (151). However, a recent study has shown that EPHB4 might function via regulating hypoxia. In A375 melanoma xenografts, EPHB4 overexpression promoted tumor growth but prevented vascularization. This effectively created a hypoxic environment, especially in small tumors. EPHB4 also increased expression of the immunosuppressant IL-8 and TGF-β2 (152, 153). In addition, TGF-β2 was shown to act as a master regulator of EMT in acidosis adapted cells, in colorectal and squamous cell carcinomas (154). Another study found EPHB4 activity inhibition in SCC resulted in attenuated TAM and Treg populations while simultaneously enhancing T cell activity (155). EPHB4 likely imparts similar effects in the melanoma immune environment, in accordance with the effects of hypoxia generation.

#### 5.1.2 Transcription factor 4

Transcription Factor 4 (TCF4) plays a role in Wnt/β-catenin signaling and is upregulated in BRAF-inhibitor resistant melanoma cells. In vemurafenib resistant cell lines, TCF4 knockdown sensitized melanoma cells to vemurafenib in a Glucose transporter 3 (GLUT3)-dependent manner, thereby decreasing lactate production. TCF4 inactivity has also been shown to be responsible for downregulation of metastatically-related genes (156). Given the acidifying role of lactate in the TME, it is unsurprising another study found TCF4 is enriched in “mesenchymal-like” cells and a suppressor of antigen-presentation programs. Targeting TCF4 in “mesenchymal-like” melanoma increased immunogenicity and yielded a targeted therapy benefit (157).

#### 5.1.3 Monocarboxylate transporters

MCTs are known to contribute to proliferation, immune escape, and are essential for glycolysis clearance (13). Diclofenac, a nonsteroidal anti-inflammatory drug (NSAID), was found to impair MCT function in a melanoma B16 mouse xenograft model. This was found to result in increased anti-PD-1 blockade efficacy, increased T cell activity during anti-PD-1 blockade, decreased lactate secretion, and increased pH in the TME. *In vitro*, upon diclofenac treatment, CD8^+^ T, CD3^+^ T, and NK cell numbers increased alongside IFNγ expression. Furthermore, diclofenac selectively disabled the cancer glycolytic shield; immune cell functions or glycolysis were not hindered to nearly the same degree (13). In patient tumor samples, the fourth MCT family member, MCT4, displays increased expression in the progression from primary to metastatic melanoma and both MCT4 and MCT1 expression predict poor patient survival (158). Inhibiting the first MCT family member, MCT1, with a small molecule inhibitor reduced circulating tumor cells in blood and overall metastatic burden in patient-derived melanoma xenografts. Notably, as NSG mice were used, this indicates a metabolic metastasis program operating separately from metastasis originated from altered immune escape capabilities (159). These recently exposed targets increase the allure of disabling acidosis and hypoxia drivers. A variety of metastatically and immunologically related benefits may be achieved by halting these progression related processes.

### 5.2 Recently revealed factors driven by hypoxia and acidosis

#### 5.2.1 Hypoxia-inducible transcription factor-1α

HIF-1 is an oxygen sensitive master regulator responsible for a vast number of the hypoxia-driven effects in melanoma and other cancers (160, 161). The HIF-1 oxygen-regulated alpha subunit (HIF-1α) is well studied in melanoma and its abilities to drive metastasis and negate immune responses or immunosurveillance (160, 162). HIF-1α has also been linked to upregulation of immune checkpoint proteins and the release of immunosuppressants including certain interleukins and TGF‐β (162). While these roles are well established, recent studies continue to update the importance of HIF-1α in melanoma immunity and metastasis.

In metastatic melanoma patients, Foxp3^+^ and Retinoic Acid Receptor-related Orphan Receptor-γ^+^ (RORγ^+^) lymphocytes were correlated with high HIF-1α expression (163). This is indicative of T helper 17 (Th17) Tregs (160–162). Th17 Tregs have been previously found to promote melanoma progression through activation of the IL-6/STAT3 pathway (164). Further, STAT3 and HIF-1α may regulate each other, potentially in self-amplifying cycles (160, 163, 165, 166). In line with these results, B16-F10 mice injected in T cell specific HIF-1α knockout mice displayed reduced growth compared to wild-type. Combinatorial treatment using the HIF-1α inhibitor Acriflavine and the Treg depletion agent Cytoxan improved CD8^+^ T cell responses and reduced tumor growth when compared to Acriflavine or Cytoxan alone (167). Another study using B16-F10 injection found tumor-specific HIF-1α deletion enhanced CD4^+^, CD8^+^, and NK cell infiltration. Acriflavine also provided benefit to an anti-PD-1 and Tyrosinase-related protein-2 (TRP2) vaccination immunotherapy combination (168).

The HIF-1α inhibitor, IDF-11774, achieved similar results in a nude mouse B16-F10 model, diminishing tumor growth and increasing CD8^+^ T cell infiltration. Additionally, IDF-11774 reduced expression of the EMT markers SNAIL and N-cadherin (169). HIF-1α regulates melanoma VEGF expression, and increasing evidence supports the HIF-1α/VEGF axis contributes to metastasis and EMT (170–172). Another emerging HIF inhibitor, 64B, diminished liver metastasis rates and volumes, and diminished lung metastasis in a 92.1 uveal melanoma model (173). Despite these findings, contradictory studies have found HIF-1α is needed for helper T cell differentiation (174), induces cytokine production programs in CD8^+^ T cells (175), and encourages NK cell effector function (176). Relatively uniform evidence backs up the immune-evasion and metastatic nature of melanoma-intrinsic HIF-1α signaling, but additional research is required to understand the context(s) altering HIF-1α function in T cell and NK cell biology.

HIF-1α can also contribute to melanoma phenotype switching. Activation of HIF-1α has been shown to stimulate glucose accumulation and induce MITF expression. However, this response is transient and under prolonged hypoxia MITF will repress itself (177). This process could contribute to intertumoral heterogeneity across hypoxia gradients in different tumor locations, and increase the prevalence of MITF^low^ populations. In line with this idea, another report demonstrated tumor acidosis reprogrammed melanoma into the MITF^low^/AXL^high^ phenotype (178). Thus, HIF-1α can indirectly, via EMT induction, or directly via MITF/AXL expression, shift melanoma phenotypes towards immunosuppressive and metastatically-inclined states. Taken together, HIF-1α plays key roles in regulating melanoma signaling responsible for EMT, and ultimately metastasis, and immune responses. Recent findings suggest a prominent role of HIF-1α in regulating lymphocyte pacification, but further clarification in this aspect is required. HIF-1α small molecule inhibitors will be pivotal for future studies in this direction and future therapeutic ventures building on our existing knowledge.

#### 5.2.2 Ovarian cancer G-protein-coupled receptor 1

OGR1 is a member of the proton-sensing G-protein-coupled receptors (GPCRs), which emit responses dependent on extracellular pH (143, 179). Past research has shown that hypoxia-driven activation of these receptors promotes survival and furthers glycolytic metabolic pathways (180). OGR1 is upregulated in melanoma and expressed by both melanoma and immune-involved cells. Depletion of OGR1 in a B16-F10 metastasis model resulted in decreased migratory colony formation. OGR1 targeting also activated T cells and improved T cell killing, stimulated IFNγ and IL-18 release, and antigen processing and presentation. T cells extracted from OGR1 deficient mice (spleen) were better adapted to acidic environments (179). Another recent study investigating OGR1, and the related GPCR T cell death-associated gene 8 (TDAG8), found these receptors mediate pH-dependent PD-L1 expression increases in B16-F10 melanoma cells. PD-L1 expression was decreased and CD8^+^ T cell infiltration improved by the addition of basic sodium bicarbonate (181). G-Protein Coupled Receptor 4 (GPR4) is another acidosis activated receptor. One study found its function is opposite to OGR1 and TDAG8; its overexpression at an acidic pH prevented migration and improved adhesion in B16-F10 cells through activation of the Rho/ROCK pathway (182). Previous studies have also shown that Rho/ROCK signaling is important in the regulation of melanoma EMT and immune responses, demonstrating how acidic-driven signals can manifest the malignant phenotype (128, 183, 184).

#### 5.2.3 Brain and muscle arnt-like protein-1

BMAL1 is a master regulator of circadian clock-related genes (185, 186). Previous studies evidence the importance of circadian rhythm-related genes in metabolic activities (186). Furthermore, BMAL1 contributes to activation of pro-survival signaling pathways in response to oxidative stress (187). In melanoma cell lines, BMAL1 activities were found to oppose those of HIF-1α and promote oxidative phosphorylation. BMAL1 macrophage knockout increased HIF-1α activity and produced more lactate. Furthermore, disruption of the glycolytic balance in macrophages through BMAL1 increased their TAM likeness. BMAL1 loss has been hypothesized as a contributor to reduced CD8^+^ T and NK cell function (186). BMAL1 is involved in both hypoxic maintenance and the cellular response to an impaired environment (186).

#### 5.2.4 Leucine-rich repeats and immunoglobulin-like domains 1

LRIG1 inhibits epidermal growth factor receptor (EGFR) signaling and is downregulated in several forms of cancer. In melanoma cell lines, hypoxia decreases LRIG1 expression, favoring EMT. LRIG1 overexpression blunted hypoxia-driven EMT and formation of vascular channels through EGFR/Extracellular Signal-Regulated Kinase (ERK) signaling inhibition (188).

#### 5.2.5 Baculoviral IAP repeat-containing 2

BIRC2 is a ubiquitin-protein ligase involved in governing proteasomal degradation and apoptosis (189–191). BIRC2 expression is activated in hypoxic conditions through multiple HIFs. In B16-F10 cells, BIRC2 expression is shown to block chemokine C-X-C motif (CXCL) 9 release, a chemokine responsible for CD8^+^ T and NK cell recruitment. In B16-F10 mice, BIRC2 knockdown also decreased T cell immunoreceptor with Ig and ITIM domains (TIGIT) and LAG-3 expression while increasing DC levels (190). Another recent study using a B16-F10 murine model found BIRC2 deletion prevented lung tumor nodule formation (191). In addition, BIRC2 has been identified as a regulator of NF-kappaB, providing a further link between BIRC2, metastasis and immune escape (192).

**Figure 2:**
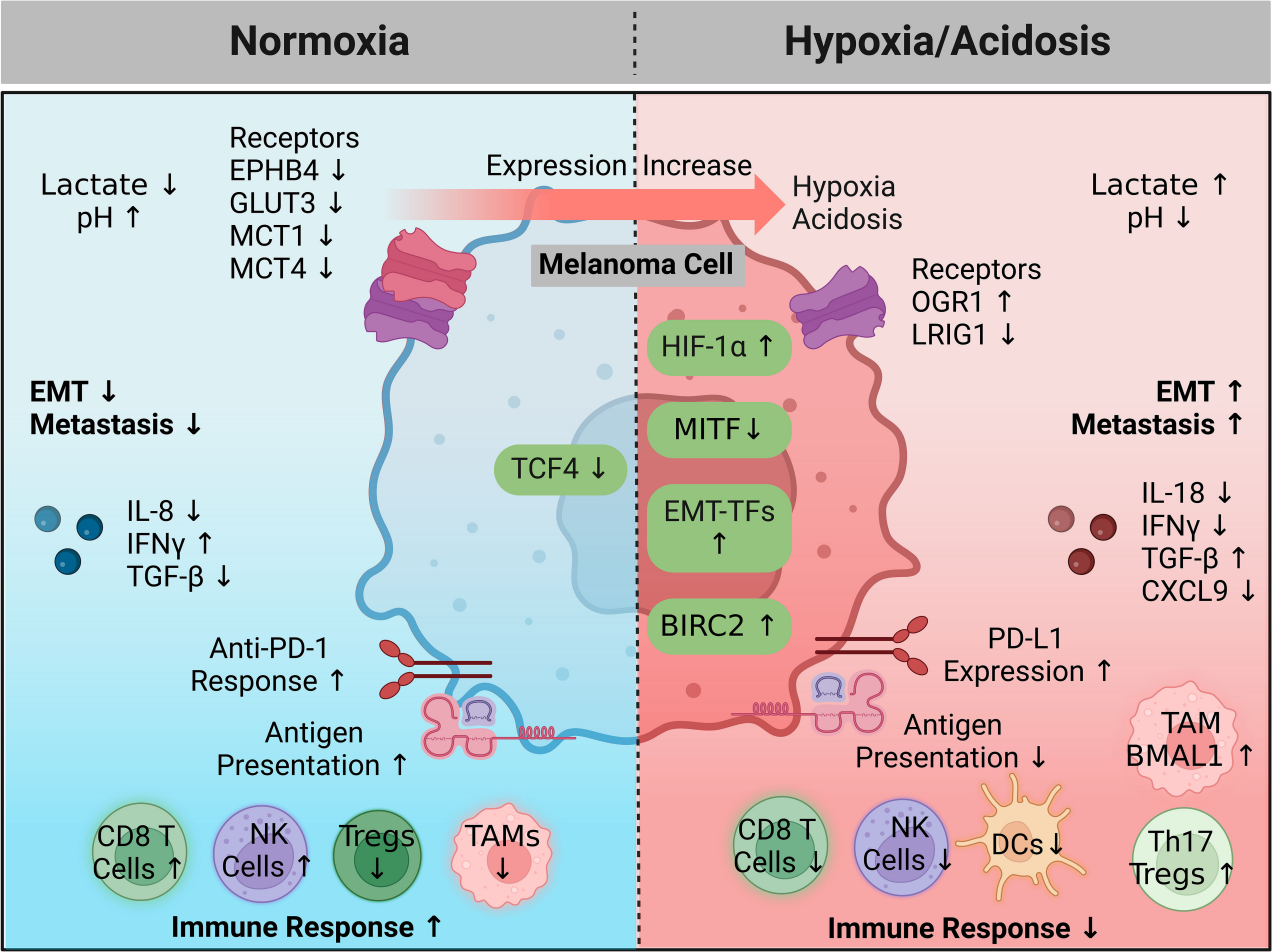
A hypoxic and acidic environment favoring immune escape and metastasis. In healthy cells HIF-1α and a microenvironment pH are tightly regulated. During melanoma progression, increased expression of key genes (hypoxia driving factors) coordinate to generate a hypoxic and acidic microenvironment that promotes immune escape, while also activating intrinsic programs enhancing metastatic capabilities. Feedback from the microenvironment can subsequently activate surface sensors or other proteins (hypoxia driven factors), resulting in amplifying feedback loops that continue this cycle. Treatment intervention in the microenvironment or targeting intrinsic signaling pathways controlling or being controlled by hypoxia may halt melanoma progression or allow immune killing. Created with BioRender.com. BIRC2, baculoviral IAP repeat-containing 2; CXCL9, chemokine (C-X-C motif) ligand 9; DC, dendritic cell; EPHB4, EPH receptor B4; EMT, epithelial to mesenchymal transition; EMT-TFs, epithelial to mesenchymal transition transcription factors; GLUT3, glucose transporter 3; HIF-1 α, hypoxia inducible factor 1 alpha; IFNγ, interferon gamma; IL, interleukin; LRIG1, leucine rich repeats and immunoglobulin like domains 1; MCT, monocarboxylate transporter; MITF, melanocyte inducing transcription factor; NK, natural killer; OGR1, ovarian cancer G protein-coupled receptor 1; PD-1, programmed cell death protein 1; PD-L1, programmed death ligand 1; TAM, tumor associated macrophage; TCF4, transcription factor 4; Treg, regulatory T cell; TGF- β, transforming growth factor beta.

Altogether, hypoxia and acidosis are key developments associated with tumor progression. Various mechanisms contribute to the generation of a hypoxic and acidic immunosuppressive microenvironment, including HIF-1α and many other targets (**Figure 2**). Signals stimulated by this environment then promote metastasis and further enable the immune-resistant properties of melanoma. The observed reversibility of pH-dependent effects and potential to break powerful regulatory feedback systems is therapeutically appealing for future melanoma treatment. Further research is required to understand how melanoma cells utilize the hypoxic mechanism to enhance their invasive abilities.

## 6 Immune escape and early dissemination

The classical, or linear, model of cancer progression predicts late-stage dissemination from the primary tumor site and subsequent initiation of metastatic colonies genetically reflective of their origin. However, recent research suggests that melanoma cells actually disseminate early to distant sites where they escape immune recognition (23, 24). These cells exist in a dormant state and can initiate metastatic colonies after signaling activation at a later time (22–24). Eyles et al. delved deeper into the concept of early tumor cell dissemination. In a RET.AAD (transgenic for the human oncogene *RET* and the mouse/human chimeric MHC antigen AAD), spontaneous melanoma mouse model, successful dissemination was observed as early as 3 weeks after primary tumor onset. 1.5 years later, metastatic colonies stemming from the initial disseminations were observed. Disseminated tumor cells were dormant for varying tissue-dependent periods of time, and this led to staggered metastatic outgrowth. The authors suggested that the dormant disseminated state arises from a flawed immune response resulting in temporary cell cycle arrest rather than apoptosis (22).

The genetic landscape of disseminated cancer cells also vastly differed from their primary site; this also supports a model favoring early dissemination as disseminated cells and primary tumor cells will have large amount of time to acquire genetic differences. In the melanoma progression model proposed by Werner-Klein et al., dissemination is an early phenomenon of melanoma in which 1) immune-evasive and malignantly inept cells seed the body, 2) disseminated cancer cells genetically differentiate, 3) stimulation triggers colony formation and switching to a proliferative phenotype (24). These results challenge the classical linear progression model and show that tumor cells may disseminate across the body early in disease progression. To prevent recurrence and develop treatments targeting dormant disseminations, the progressive mechanisms promoting early dissemination and dormant melanoma states must be further researched. Here, we summarize recent studies providing novel evidence in this area.

### 6.1 Hypoxia vs oxidative phosphorylation in early dissemination and dormancy

The shift between hypoxia-induced metabolic shifts and initially oxidatively sound states may provide insight into the mechanisms allowing dissemination survival and dormancy. Past research indicates dormant melanoma cells exist in a stem cell-like state (193). In a murine model, mouse granulocyte-macrophage colony-stimulating factor expressing B16-F1 cells were used to develop immune-resistant dormant disseminations in various organs while suppressing primary tumor development. These quiescent populations lacked proliferative ability and expressed the melanoma stem cell markers CD133 and CD24. Their enhanced self-renewal properties compared to maternal specimens also indicates an increased likeness to melanoma stem cells. Furthermore, Glucocorticoid-induced Leucine Zipper (GILZ) was identified as a regulator of the G0-to-G1 transition in these dormant populations with quiescent cells lacking GILZ expression. In a human melanoma model, GILZ downregulation promoted the development of dormant stem-cell like states through FOXO3a activation, increased G0 cells, promoted tumorigenicity, and decreased expression of the differentiation marker tyrosinase. This provides evidence that beyond previously described immune-selection models, intrinsic pathways inhibiting differentiation, such as GILZ depletion, may develop stem-like states capable of producing dormant and highly tumorigenic seeds (193).

The role of hypoxia (or lack of in this case) in dormancy becomes more apparent through FOXO3a, which was recently found to promote Sirtuin 6 (SIRT6) expression. SIRT6 then represses Myc and HIF-1α while simultaneously deacetylating glycolysis promoting genes (194). The role of SIRT6 in melanoma proliferation or dormancy requires further investigation, as past studies have shown a pro-proliferative role unsupportive of dormant states (195, 196). This indicates FOXO3a serves as a glycolytic suppressor; it is then likely that in the GILZ downregulated melanoma models oxidative phosphorylation prevails, although this remains unknown. In melanoma cells, oxidative phosphorylation has been shown to be critical in maintaining quiescence (197). In another recent report, quiescent melanoma cell lines were identified by high and low p27 and Ki67 expression. Interestingly, oxidative phosphorylation was conserved, and even enhanced, in dormant cells. Oxidation inhibition promoted the return to cycling cell states. In the dormant cell populations, cellular myelocytomatosis oncogene (c-Myc) was overexpressed and identified as a potent driver of oxidative phosphorylation pathways (198). When taken with this possible role of FOXO3a in the GILZ models, it becomes apparent that maintenance of oxidative phosphorylation may be a conserved mechanism across dormant, stem-like states with high tumor initiation potential.

### 6.2 Proliferation inducing pathways in early dissemination and dormancy

Studies have suggested that inhibition of PI3K/AKT signaling deactivates the cell cycle in melanoma stem cells and promotes dormancy (199). The PI3K/AKT pathway is also a downstream target of GILZ, and PI3K/AKT activation halts FOXO3a activity (193). In metastatic melanoma cell lines, PI3K/AKT inhibition switched proliferative subsets to slower cycling stem cell like states. Interestingly, this switch was accompanied by reduced HIF-1α levels, however HIF-1α was still overexpressed in the stem-like populations (199). One recent study found 5′AMP‐activated protein kinase (AMPK) expression is increased in c-Myc reliant melanoma cells (200). AMPK promoted c-Myc^+^ survival in melanoma mouse models (200). AMPK functions as a buffer against oxidative stress and stimulates catabolic ATP generation through oxidative phosphorylation maximization (201, 202). AMPK also acts to inhibit PI3K/AKT signaling (200). From these observations, it seems a tight balance between oxidative phosphorylation maintenance and PI3K/AKT signaling is necessary for sustaining dormancy.

**Figure 3:**
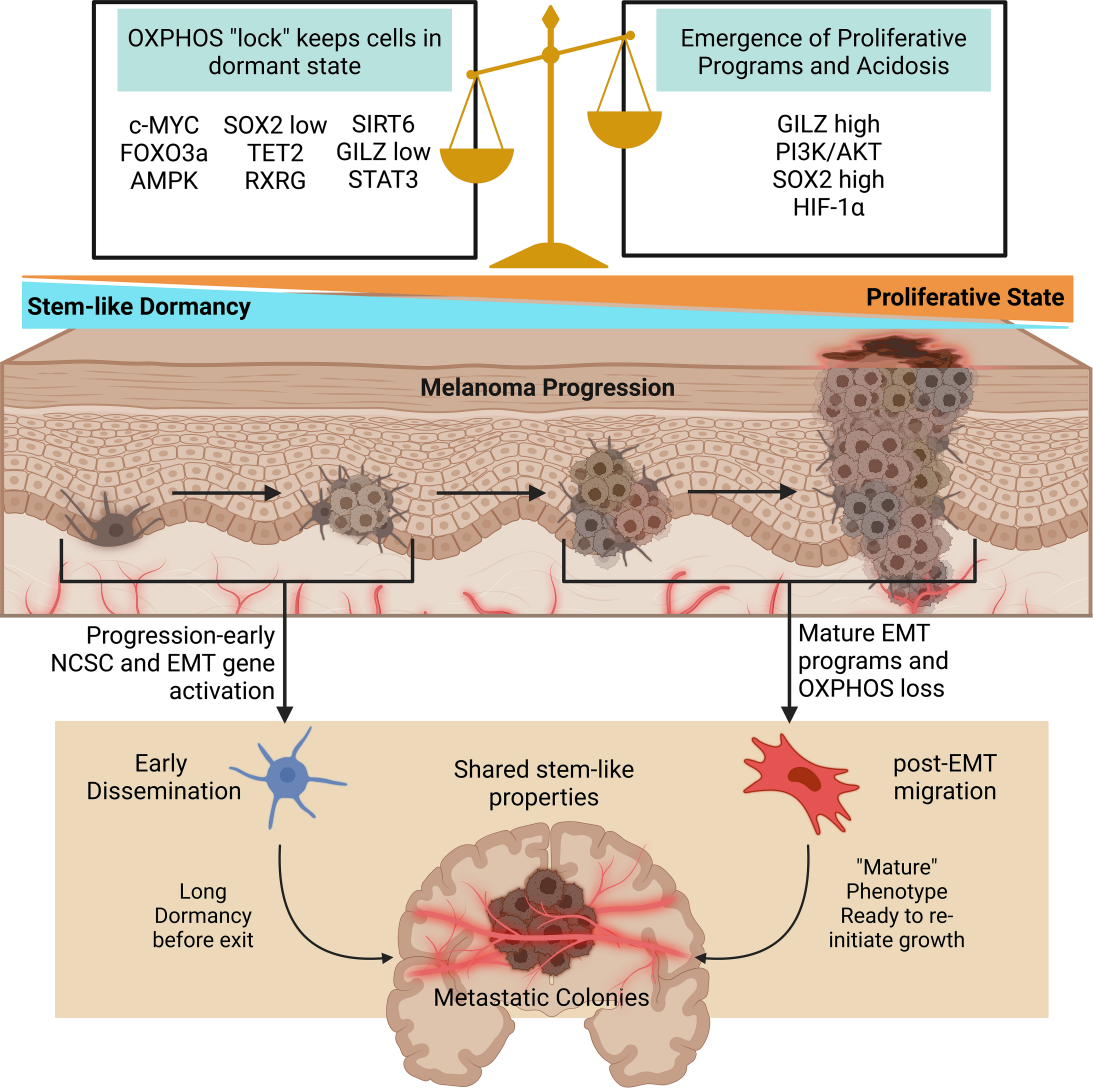
Oxidative phosphorylation and stem pathways govern early dissemination. As dormant stem-like cells face further dysregulation and heterogenization proliferative programs and acidosis replace the delicate balance initially allowing early dissemination. In both cases, metastatic colonies are formed with similar stem-like properties. Early disseminated cells remain dormant for long periods of time, and the mechanisms causing growth re-initiation remain to be well studied. Created with BioRender.com. AMPK, 5′AMP‐activated protein kinase; c-MYC, myc; EMT, epithelial to mesenchymal transition; FOXO3a, forkhead box O3; GILZ, glucocorticoid-induced leucine zipper; HIF-1α, hypoxia inducible factor 1 alpha; OXPHOS, oxidative phosphorylation; RXRG, retinoid X receptor gamma; SIRT6, sirtuin 6; SOX2, SRY-box transcription Factor 2; STAT3, signal transducer and activator of transcription 3; TET2, tet methylcytosine dioxygenase 2.

In a recent model utilizing tumorigenic melanoma tumor-repopulating cells, high SOX2 (a member of the SOX transcription factors) levels fueled growth, low levels of SOX2 put cells into a state of dormancy, and a complete knockout of SOX2 exited the quiescent state into a less stem-like phenotype (197). Another recent report also finds SOX2 contributes to enhanced oxidative phosphorylation in melanoma (203). However, acidosis accumulation promotes stemness factors including SOX2 (139). Based on these reports, a progression-related hypoxia and acidosis model of early dissemination may be proposed. In early tumor formation, severe hypoxia and acidosis are not present. The key to early dissemination may rely on activation of factors promoting stemness without deterioration of oxidative phosphorylation (**Figure 3**). As proliferation inducing pathways like PI3K/AKT are further stimulated, this balance promoting dormancy is lost and replaced with proliferation. Hypoxic conditions may still work to promote EMT and metastatic phenotypes, but through different genetic programs from the early oxidatively enhanced disseminations.

### 6.3 Neural crest stem cell related genes and EMT overlap in early dissemination and dormancy

Genes important for NCSCs that contribute to EMT are also emerging as contributors to dormancy. SOX2 is important to neural crest development but in melanoma it is a contributor to stemness and invasive ability (204, 205). High Tet Methylcytosine Dioxygenase 2 (TET2) expression was observed in multiple slow cycling dormant-like tumor subsets. Tet family proteins have been implicated in embryonic development and the neural lineage (206). In a recent study using a melanoma mouse model, TET2 expression promoted suppressive TAM genetic profiles and suppressive myeloid cell activities (207). In another melanoma mouse model, TET2 depletion increased AKT and proliferation marker expression, decreased cell cycle inhibitors, and accelerated tumor development (208).

Another study utilized MAPK-inhibition to develop quiescent NCSC-like melanoma populations. The neural crest migration gene, Retinoid X Receptor Gamma (RXRG), was overexpressed in these cells and identified as a driver of NCSC states (135, 209). IFN-β can also act as an effective inducer of dormancy, this is partially attributed to its ability to induce STAT3 serine phosphorylation. In this dormancy model, dual serine tyrosine phosphorylation activated p53 to promote apoptosis (210). In the neural crest, STAT3 regulates cell cycle progression and differentiation (211). More research is required to understand what genetic prerequisites control proliferative or dormant stem-like outcomes in NCSC genes. While many genes important for NCSC maintenance have been linked with EMT and stemness properties, their roles in dormancy maintenance remain a lacking area of study.

In a recent study, Rapanotti and colleagues isolated circulating melanoma cells from patients in varying stages of disease progression. In early-stage patients, the authors utilized three subsets of circulating cells including endothelial-like (CD45^-^ MCAM^+^), stem-like (CD45^-^ ABCB5^+^), and hybrid-like (CD45^-^ MCAM^+^ ABCB5^+^). The endothelial population expressed adhesive markers, but all three populations were found to exhibit “stem-mesenchymal” like characteristics (212). Through NCSC genes and EMT, mesenchymal and stem-like traits are conferred to melanoma cells. This indicates that the same NCSC genetic programs hijacked to drive EMT, may also drive early dissemination as these cells display similar properties. A recent study examining transcriptomes from melanoma cells found EMT/invasive states and NCSC-like cells differ in their genetic backgrounds, but both exist with low levels of MITF (135). These findings support the idea of early progression NCSC genetic manipulation and are in line with the stem-mesenchymal properties of circulating melanoma cells. This is also supported by various recently discovered roles of NCSC genetic programs in promoting dormancy. It is likely that initial tumorigenic cells possess high levels of stemness, allowing early dissemination to occur before later progression-related changes and heterogenization blunt the delicate genetic balance held in these cells. Late MITF^low^ populations formed from EMT may reflect early dissemination, however, varying mechanisms will have led to their final stem-like states. The same drivers promoting metastasis and immune escape through EMT likely act as initial stemness conveyors responsible for early dissemination as tumors are initiated in a pre-hypoxic environment. Overall, understanding the properties of early disseminated or dormant cells as well as the mechanism behind their formation is critical for reducing melanoma recurrence. More NCSC markers and EMT programs need to be studied in the context of early tumor progression and initiation so that novel treatments to maintain/lock the stem state dormancy may be developed.

## 7 Therapeutic opportunities against immune escape, EMT and early dissemination

**Figure 4:**
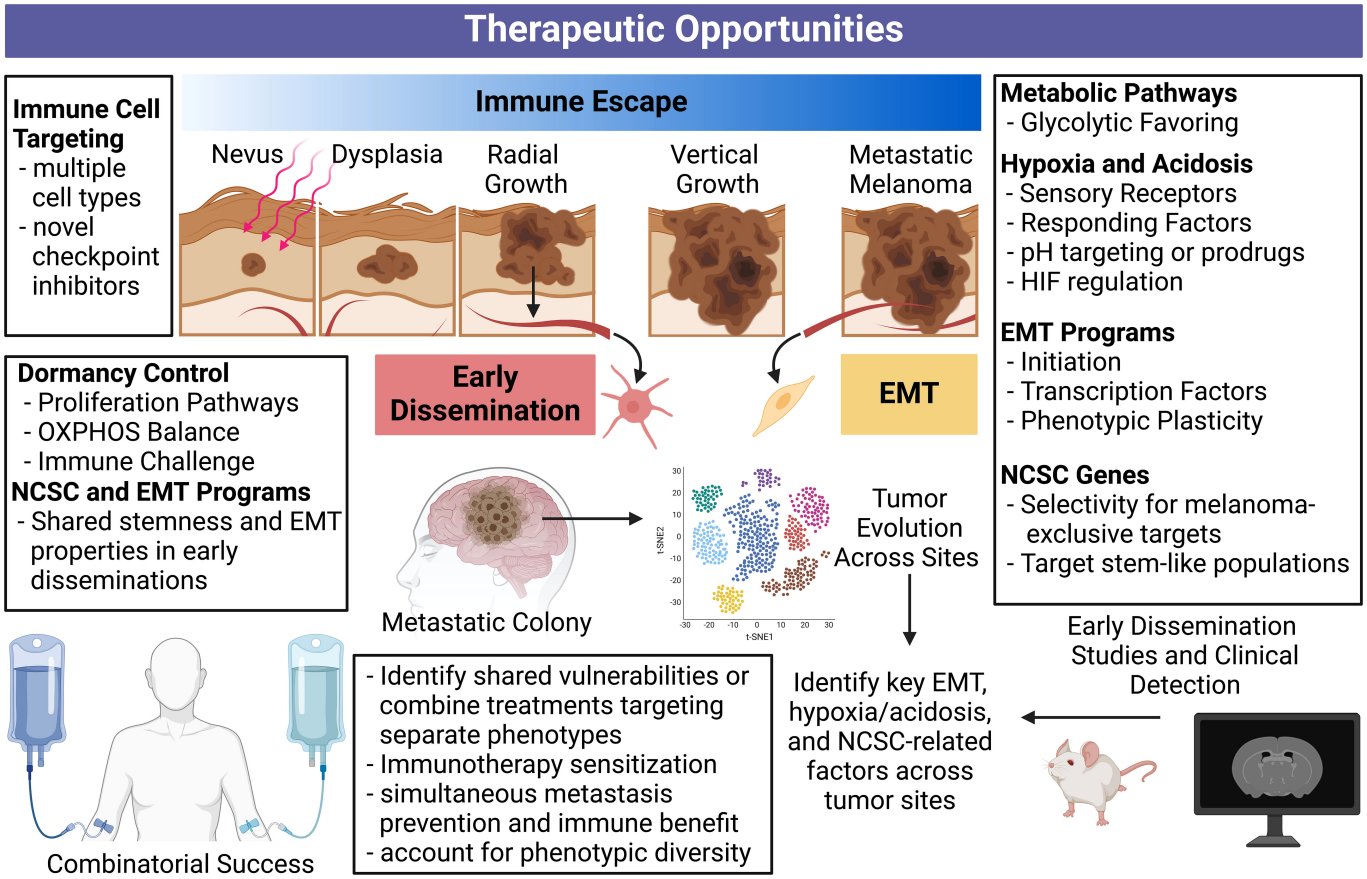
Comprehensive overview of therapeutic opportunities against immune escape, metastasis, and early dissemination. Targeting processes concurrently driving immune escape and metastasis is therapeutically appealing and may lead to synergistic treatments. Disrupting these processes can simultaneously halt multiple fronts of melanoma progression. Additionally, applying our understanding of early dissemination to future bioinformatic tools and long-term mouse models may reveal more specific vulnerabilities and detection methods for these slow and silent killers. An enhanced understanding of stemness programs and phenotype plasticity can help deter therapeutic resistance. Our current understanding of immune escape, metastasis, and early dissemination can inform the future studies required to revolutionize metastatic melanoma treatment. Created with BioRender.com. NCSC, neural crest stem cell; OXPHOS, oxidative phosphorylation.

“Dual drivers” concomitantly contributing to immune escape and metastasis are alluring therapeutic targets with the potential to sensitize melanoma cells to immune attack while simultaneously preventing disease progression. We have summarized therapeutic opportunities and future directions related to this concept in **Figure 4**.

Targeting EMT, either through pathways enabling the EMT process, or characteristics of dedifferentiated post-EMT phenotypes, is an alluring approach to manage melanoma progression. However, tumor heterogeneity is a well-known contributor of therapy resistance as reviewed in (213), making the EMT process or products difficult targets. Applying tumor evolution algorithms and looking for shared markers across sub-clonal tumor populations may be able to overcome this problem in a search for shared vulnerabilities. Alternatively, therapies directed at different phenotypes could be employed in combination, or sequentially, to account for EMT-induced phenotypic diversity.

Ectopic expression of NCSC-relevant genes in melanoma may also provide therapeutic opportunity, as normal melanocytes and many other healthy tissues do not express the proteins coded by these genes. Additionally, expression of NCSC markers can contribute to the EMT process, and immune escape and metastasis through separate means. The same can be said for hypoxia and acidosis driven or driver targets. In this case, pH may be an opportunity to promote selectivity or deliver therapies. As pseudohypoxia, or the induction of hypoxia-related genes despite sufficient oxygen (177), is present in a wide-range of melanomas, hypoxia-related genes could maintain therapeutic potential in a wide range of patients. Overall, EMT initiators/products, NCSC-relevant targets, and the hypoxic response serve as points of therapeutic interference with potential to simultaneously enhance melanoma immunity and prevent metastatic progression. The expansion of single-cell RNA sequencing techniques and vulnerability screens can better inform what targets in these processes are most relevant, shared between subclones within tumors, and even between tumors after metastatic progression.

The role of early dissemination in contributing to clinically presenting metastatic melanoma remains to be well documented. The hypoxic properties characteristic of late-stage primary tumors contrasts the potential oxidative phosphorylation favoring in dormant disseminations, possibly complicating a shared therapeutic approach focused on metabolism. However, both cases display overlap in NCSC-relevant genes and EMT features. Additional early dissemination mouse studies and parallel studies using clinical specimens can demonstrate differences in secondary tumors arising from early dissemination versus late-stage primary spread. Separate therapeutic approaches may be required in these cases, potentially prolonging dormancy or preventing dormancy exit if dormant disseminations cannot be destroyed.

It is worth noting that melanoma brain metastasis (MBM) remains a critical clinical problem in melanoma management. Further, 20-40% of melanoma patients experience brain metastasis, with a median overall survival of only 8.9 months after diagnosis (214). As an immune privileged site, MBM experience a unique TME vastly different from other metastatic sites, characterized by resident microglia immune cells that play a similar role to macrophages, the central nervous system (CNS) constituents, such as astrocytes and neurons, high vascular density, extracellular matrix differences, and a blood-brain barrier that must be compromised to allow robust immune infiltration. These factors can combine to generate a deeply immunosuppressive environment that prevents success of current therapies (as reviewed in (215)). The MBM TME can also drive melanoma progression through unique crosstalk events. For example, melanoma cells can alter astrocytes to promote monocyte chemoattractant protein-1 (MCP-1) production, which in turn drives MBM proliferation and invasion (216). Microglial JAK/STAT activation from melanoma-secreted IL-6 can similarly enhance MBM progression (217).

MBM shares some of the metastatic features discussed above, such as increased AXL and a NGFR^high^ dedifferentiated state (214, 218). In the brain, dedifferentiation is shown to be enhanced by astrocyte or microglia-derived cytokines such as TGF-β. Similarly to the concept of reactivating dormant programs important for neural crest stem cell function, MBM cells can easily enter a neuronal-like state, which has been proposed to enhance brain-melanoma compatibility. However, more differentiated MBM E-cadherin^high^ states have also been identified, potentially dependent on high oxidative phosphorylation (214). Indeed, in sharp contrast with the pro-metastatic role of hypoxia, oxidative phosphorylation targeting in MBM-bearing mice greatly improved survival (219), and MBM oxidative phosphorylation was shown to be associated with worse immune cell infiltration (220). However, a recent spatial single-cell transcriptomics study showed heterogeneous expression of oxidative phosphorylation in treatment-naive MBM (218). MBM oxidative phosphorylation variance could reflect observations in cases of early dissemination or dormancy and represent stem-like states with high tumorigenic potential, as described above. Just as separate therapeutic approaches may be needed to successfully tackle early dissemination-derived metastasis, a similar, multifaceted, approach may be needed to conquer the unique brain TME.

As targeting the EMT, NCSC, or hypoxia/acidosis features of melanoma will enhance melanoma immune responses, combinations with current and upcoming immunotherapies should be considered. Future research accounting for patient-specific, or interpatient, heterogeneity can answer which features in these programs are most therapeutically appealing should be conducted. Additionally, differences between early dissemination arising secondary tumors and those arising late in disease progression must be investigated. Answering these questions in the context of EMT, NCSC, and hypoxia/acidosis related genes will provide therapeutic opportunities with the potential to augment melanoma immunotherapy responses while preventing future metastatic recurrence.

## 8 Conclusion

Melanoma immunotherapies have dramatically improved patient treatment and survival, yet immunotherapy resistance and disease recurrence remain prevalent. Shared mechanisms simultaneously governing melanoma immune evasion and metastases potentially influence both of these barriers, preventing successful treatment. Specifically, the EMT process, genes involved in NCSC maintenance, and hypoxia/acidosis contribute to both immune evasion and metastatic development during melanoma progression. These processes may also exert a relevant influence in early dissemination. In the future, therapeutic focus may benefit from examining dual drivers of immune evasion and metastasis. Single cell transcriptomic studies focused on heterogeneity and tumor evolution trajectories can reveal what factors relevant to EMT, re-activated NCSC signaling, or hypoxia/acidosis are most applicable, robust, and specific, across patients. A better understanding of these factors will result in future combinational approaches that take advantage of current immunotherapies to prevent melanoma recurrence and resistance.

## Author contributions

CS: Conceptualization, Writing – original draft, Writing – review & editing. GC: Conceptualization, Writing – original draft, Writing – review & editing. DA: Writing – original draft. HC: Writing – review & editing. NA: Conceptualization, Writing – review & editing, Supervision.

## References

[1] Carlino MS Larkin J Long GV. Immune checkpoint inhibitors in melanoma. Lancet (2021) 398(10304):1002–14. doi: 10.1016/S0140-6736(21)01206-X 34509219

[2] Switzer B Puzanov I Skitzki JJ Hamad L Ernstoff MS. Managing metastatic melanoma in 2022: A clinical review. JCO Oncol Pract (2022) 18(5):335–51. doi: 10.1200/OP.21.00686 PMC9810138 35133862

[3] Atkins MB Curiel-Lewandrowski C Fisher DE Swetter SM Tsao H Aguirre-Ghiso JA. The state of melanoma: emergent challenges and opportunities. Clin Cancer Res (2021) 27(10):2678–97. doi: 10.1158/1078-0432.CCR-20-4092 PMC8127340 33414132

[4] Luan W Ding Y Yuan H Ma S Ruan H Wang J. Long non-coding rna linc00520 promotes the proliferation and metastasis of Malignant melanoma by inducing the Mir-125b-5p/Eif5a2 axis. J Exp Clin Cancer Res (2020) 39(1):96. doi: 10.1186/s13046-020-01599-7 32466797 PMC7254730

[5] Brito C Barral DC Pojo M. Subversion of ras small gtpases in cutaneous melanoma aggressiveness. Front Cell Dev Biol (2020) 8:575223. doi: 10.3389/fcell.2020.575223 33072757 PMC7538714

[6] Zhang Y Hou J Shi S Du J Liu Y Huang P. Csn6 promotes melanoma proliferation and metastasis by controlling the Ubr5-mediated ubiquitination and degradation of Cdk9. Cell Death Dis (2021) 12(1):118. doi: 10.1038/s41419-021-03398-0 33483464 PMC7822921

[7] Yang Y Zhe H Massoumi R Ke H. Decreased expression of nemo-like kinase in melanoma is correlated with increased vascularity and metastasis. Melanoma Res (2019) 29(4):376–81. doi: 10.1097/CMR.0000000000000576 30778016

[8] Sun X Cao N Mu L Cao W. Stress induced phosphoprotein 1 promotes tumor growth and metastasis of melanoma *via* modulating Jak2/Stat3 pathway. BioMed Pharmacother (2019) 116:108962. doi: 10.1016/j.biopha.2019.108962 31103826

[9] Hamid O Robert C Daud A Hodi FS Hwu WJ Kefford R. Five-year survival outcomes for patients with advanced melanoma treated with pembrolizumab in Keynote-001. Ann Oncol (2019) 30(4):582–8. doi: 10.1093/annonc/mdz011 PMC6503622 30715153

[10] Valentin J Ferte T Dorizy-Vuong V Dousset L Prey S Dutriaux C. Real-world survival in patients with metastatic melanoma after discontinuation of anti-Pd-1 immunotherapy for objective response or adverse effects: A retrospective study. J Oncol (2021) 2021:5524685. doi: 10.1155/2021/5524685 33995528 PMC8096591

[11] Imbert C Montfort A Fraisse M Marcheteau E Gilhodes J Martin E. Resistance of melanoma to immune checkpoint inhibitors is overcome by targeting the sphingosine kinase-1. Nat Commun (2020) 11(1):437. doi: 10.1038/s41467-019-14218-7 31974367 PMC6978345

[12] Seth R Agarwala SS Messersmith H Alluri KC Ascierto PA Atkins MB. Systemic therapy for melanoma: asco guideline update. J Clin Oncol (2023) 41(30):4794–820. doi: 10.1200/JCO.23.01136 37579248

[13] Renner K Bruss C Schnell A Koehl G Becker HM Fante M. Restricting glycolysis preserves T cell effector functions and augments checkpoint therapy. Cell Rep (2019) 29(1):135–50 e9. doi: 10.1016/j.celrep.2019.08.068 31577944

[14] Boshuizen J Vredevoogd DW Krijgsman O Ligtenberg MA Blankenstein S de Bruijn B. Reversal of pre-existing ngfr-driven tumor and immune therapy resistance. Nat Commun (2020) 11(1):3946. doi: 10.1038/s41467-020-17739-8 32770055 PMC7414147

[15] Seyfried TN Huysentruyt LC. On the origin of cancer metastasis. Crit Rev Oncog (2013) 18(1-2):43–73. doi: 10.1615/critrevoncog.v18.i1-2.40 23237552 PMC3597235

[16] Wang M Xu Y Wen GZ Wang Q Yuan SM. Rapamycin suppresses angiogenesis and lymphangiogenesis in melanoma by downregulating Vegf-a/Vegfr-2 and Vegf-C/Vegfr-3 expression. Onco Targets Ther (2019) 12:4643–54. doi: 10.2147/OTT.S205160 PMC6580124 31354297

[17] Wang N Zhang H Cui X Ma C Wang L Liu W. Runx3 induces a cell shape change and suppresses migration and metastasis of melanoma cells by altering a transcriptional profile. Int J Mol Sci (2021) 22(4):2219. doi: 10.3390/ijms22042219 33672337 PMC7926509

[18] Jessen C Kress JKC Baluapuri A Hufnagel A Schmitz W Kneitz S. The transcription factor Nrf2 enhances melanoma Malignancy by blocking differentiation and inducing cox2 expression. Oncogene (2020) 39(44):6841–55. doi: 10.1038/s41388-020-01477-8 PMC7605435 32978520

[19] Aladowicz E Granieri L Marocchi F Punzi S Giardina G Ferrucci PF. Shcd binds dock4, promotes ameboid motility and metastasis dissemination, predicting poor prognosis in melanoma. Cancers (Basel) (2020) 12(11):3366. doi: 10.3390/cancers12113366 33202906 PMC7696252

[20] Rodriguez-Hernandez I Maiques O Kohlhammer L Cantelli G Perdrix-Rosell A Monger J. Wnt11-fzd7-daam1 signalling supports tumour initiating abilities and melanoma amoeboid invasion. Nat Commun (2020) 11(1):5315. doi: 10.1038/s41467-020-18951-2 33082334 PMC7575593

[21] Gofrit ON Gofrit B Roditi Y Popovtzer A Frank S Sosna J. Patterns of metastases progression- the linear parallel ratio. PloS One (2022) 17(9):e0274942. doi: 10.1371/journal.pone.0274942 36129954 PMC9491615

[22] Eyles J Puaux AL Wang X Toh B Prakash C Hong M. Tumor cells disseminate early, but immunosurveillance limits metastatic outgrowth, in a mouse model of melanoma. J Clin Invest (2010) 120(6):2030–9. doi: 10.1172/JCI42002 PMC2877955 20501944

[23] Rocken M. Early Tumor Dissemination, but Late Metastasis: Insights into Tumor Dormancy. J Clin Invest (2010) 120(6):1800–3. doi: 10.1172/JCI43424 PMC2877965 20501952

[24] Werner-Klein M Scheitler S Hoffmann M Hodak I Dietz K Lehnert P. Genetic alterations driving metastatic colony formation are acquired outside of the primary tumour in melanoma. Nat Commun (2018) 9(1):595. doi: 10.1038/s41467-017-02674-y 29426936 PMC5807512

[25] Tang Y Durand S Dalle S Caramel J. Emt-inducing transcription factors, drivers of melanoma phenotype switching, and resistance to treatment. Cancers (Basel) (2020) 12(8):2154. doi: 10.3390/cancers12082154 32759677 PMC7465730

[26] Wang G Xu D Zhang Z Li X Shi J Sun J. The pan-cancer landscape of crosstalk between epithelial-mesenchymal transition and immune evasion relevant to prognosis and immunotherapy response. NPJ Precis Oncol (2021) 5(1):56. doi: 10.1038/s41698-021-00200-4 34158591 PMC8219790

[27] Terry S Savagner P Ortiz-Cuaran S Mahjoubi L Saintigny P Thiery JP. New insights into the role of emt in tumor immune escape. Mol Oncol (2017) 11(7):824–46. doi: 10.1002/1878-0261.12093 PMC5496499 28614624

[28] Datar I Schalper KA. Epithelial-mesenchymal transition and immune evasion during lung cancer progression: the chicken or the egg? Clin Cancer Res (2016) 22(14):3422–4. doi: 10.1158/1078-0432.CCR-16-0336 PMC4947415 27076625

[29] Lotsberg ML Rayford A Thiery JP Belleggia G D’Mello Peters S Lorens JB. Decoding cancer’s camouflage: epithelial-mesenchymal plasticity in resistance to immune checkpoint blockade. Cancer Drug Resist (2020) 3(4):832–53. doi: 10.20517/cdr.2020.41 PMC8992561 35582229

[30] Taki M Abiko K Ukita M Murakami R Yamanoi K Yamaguchi K. Tumor immune microenvironment during epithelial-mesenchymal transition. Clin Cancer Res (2021) 27(17):4669–79. doi: 10.1158/1078-0432.CCR-20-4459 33827891

[31] Gonzalez H Hagerling C Werb Z. Roles of the immune system in cancer: from tumor initiation to metastatic progression. Genes Dev (2018) 32(19-20):1267–84. doi: 10.1101/gad.314617.118 PMC6169832 30275043

[32] Passarelli A Mannavola F Stucci LS Tucci M Silvestris F. Immune system and melanoma biology: A balance between immunosurveillance and immune escape. Oncotarget (2017) 8(62):106132–42. doi: 10.18632/oncotarget.22190 PMC5739707 29285320

[33] Maeurer MJ Gollin SM Martin D Swaney W Bryant J Castelli C. Tumor escape from immune recognition: lethal recurrent melanoma in a patient associated with downregulation of the peptide transporter protein tap-1 and loss of expression of the immunodominant Mart-1/Melan-a antigen. J Clin Invest (1996) 98(7):1633–41. doi: 10.1172/JCI118958 PMC507597 8833913

[34] Failli A Legitimo A Orsini G Romanini A Consolini R. Numerical defect of circulating dendritic cell subsets and defective dendritic cell generation from monocytes of patients with advanced melanoma. Cancer Lett (2013) 337(2):184–92. doi: 10.1016/j.canlet.2013.05.013 23684927

[35] Jordan KR Amaria RN Ramirez O Callihan EB Gao D Borakove M. Myeloid-derived suppressor cells are associated with disease progression and decreased overall survival in advanced-stage melanoma patients. Cancer Immunol Immunother (2013) 62(11):1711–22. doi: 10.1007/s00262-013-1475-x PMC4176615 24072401

[36] Lakshmikanth T Burke S Ali TH Kimpfler S Ursini F Ruggeri L. Ncrs and dnam-1 mediate nk cell recognition and lysis of human and mouse melanoma cell lines *in vitro* and *in vivo* . J Clin Invest (2009) 119(5):1251–63. doi: 10.1172/JCI36022 PMC2673866 19349689

[37] Munn DH Mellor AL. Indoleamine 2,3-dioxygenase and tumor-induced tolerance. J Clin Invest (2007) 117(5):1147–54. doi: 10.1172/JCI31178 PMC1857253 17476344

[38] Xing S Ferrari de Andrade L. Nkg2d and mica/B shedding: A ‘Tag game’ between Nk cells and Malignant cells. Clin Transl Immunol (2020) 9(12):e1230. doi: 10.1002/cti2.1230 PMC7754731 33363734

[39] Simiczyjew A Dratkiewicz E Mazurkiewicz J Zietek M Matkowski R Nowak D. The influence of tumor microenvironment on immune escape of melanoma. Int J Mol Sci (2020) 21(21):8359. doi: 10.3390/ijms21218359 33171792 PMC7664679

[40] Dratkiewicz E Simiczyjew A Mazurkiewicz J Zietek M Matkowski R Nowak D. Hypoxia and extracellular acidification as drivers of melanoma progression and drug resistance. Cells (2021) 10(4):862. doi: 10.3390/cells10040862 33918883 PMC8070386

[41] Dillon BJ Prieto VG Curley SA Ensor CM Holtsberg FW Bomalaski JS. Incidence and distribution of argininosuccinate synthetase deficiency in human cancers: A method for identifying cancers sensitive to arginine deprivation. Cancer (2004) 100(4):826–33. doi: 10.1002/cncr.20057 14770441

[42] Kim SH Roszik J Grimm EA Ekmekcioglu S. Impact of L-arginine metabolism on immune response and anticancer immunotherapy. Front Oncol (2018) 8:67. doi: 10.3389/fonc.2018.00067 29616189 PMC5864849

[43] Zhong X Tumang JR Gao W Bai C Rothstein TL. Pd-L2 expression extends beyond dendritic cells/macrophages to B1 cells enriched for V(H)11/V(H)12 and phosphatidylcholine binding. Eur J Immunol (2007) 37(9):2405–10. doi: 10.1002/eji.200737461 17683117

[44] Keir ME Butte MJ Freeman GJ Sharpe AH. Pd-1 and its ligands in tolerance and immunity. Annu Rev Immunol (2008) 26:677–704. doi: 10.1146/annurev.immunol.26.021607.090331 18173375 PMC10637733

[45] Jiang X Wang J Deng X Xiong F Ge J Xiang B. Role of the tumor microenvironment in Pd-L1/Pd-1-mediated tumor immune escape. Mol Cancer (2019) 18(1):10. doi: 10.1186/s12943-018-0928-4 30646912 PMC6332843

[46] Chapon M Randriamampita C Maubec E Badoual C Fouquet S Wang SF. Progressive upregulation of pd-1 in primary and metastatic melanomas associated with blunted tcr signaling in infiltrating T lymphocytes. J Invest Dermatol (2011) 131(6):1300–7. doi: 10.1038/jid.2011.30 21346771

[47] Patsoukis N Wang Q Strauss L Boussiotis VA. Revisiting the pd-1 pathway. Sci Adv (2020) 6(38). doi: 10.1126/sciadv.abd2712 PMC7500922 32948597

[48] Walunas TL Lenschow DJ Bakker CY Linsley PS Freeman GJ Green JM. Ctla-4 can function as a negative regulator of T cell activation. Immunity (1994) 1(5):405–13. doi: 10.1016/1074-7613(94)90071-x 7882171

[49] Crespo J Sun H Welling TH Tian Z Zou W. T cell anergy, exhaustion, senescence, and stemness in the tumor microenvironment. Curr Opin Immunol (2013) 25(2):214–21. doi: 10.1016/j.coi.2012.12.003 PMC3636159 23298609

[50] Das M Zhu C Kuchroo VK. Tim-3 and its role in regulating anti-tumor immunity. Immunol Rev (2017) 276(1):97–111. doi: 10.1111/imr.12520 28258697 PMC5512889

[51] Woo SR Turnis ME Goldberg MV Bankoti J Selby M Nirschl CJ. Immune inhibitory molecules Lag-3 and Pd-1 synergistically regulate T-cell function to promote tumoral immune escape. Cancer Res (2012) 72(4):917–27. doi: 10.1158/0008-5472.CAN-11-1620 PMC3288154 22186141

[52] Lines JL Sempere LF Broughton T Wang L Noelle R. Vista is a novel broad-spectrum negative checkpoint regulator for cancer immunotherapy. Cancer Immunol Res (2014) 2(6):510–7. doi: 10.1158/2326-6066.CIR-14-0072 PMC4085258 24894088

[53] Merelli B Massi D Cattaneo L Mandala M. Targeting the Pd1/Pd-L1 axis in melanoma: biological rationale, clinical challenges and opportunities. Crit Rev Oncol Hematol (2014) 89(1):140–65. doi: 10.1016/j.critrevonc.2013.08.002 24029602

[54] Lines JL Pantazi E Mak J Sempere LF Wang L O’Connell S. Vista is an immune checkpoint molecule for human T cells. Cancer Res (2014) 74(7):1924–32. doi: 10.1158/0008-5472.CAN-13-1504 PMC3979527 24691993

[55] Kalluri R Weinberg RA. The basics of epithelial-mesenchymal transition. J Clin Invest (2009) 119(6):1420–8. doi: 10.1172/JCI39104 PMC2689101 19487818

[56] Tang X Sui X Weng L Liu Y. Snail1: linking tumor metastasis to immune evasion. Front Immunol (2021) 12:724200. doi: 10.3389/fimmu.2021.724200 34917071 PMC8669501

[57] Li FZ Dhillon AS Anderson RL McArthur G Ferrao PT. Phenotype switching in melanoma: implications for progression and therapy. Front Oncol (2015) 5:31. doi: 10.3389/fonc.2015.00031 25763355 PMC4327420

[58] Hodorogea A Calinescu A Antohe M Balaban M Nedelcu RI Turcu G. Epithelial-mesenchymal transition in skin cancers: A review. Anal Cell Pathol (Amst) (2019) 2019:3851576. doi: 10.1155/2019/3851576 31934531 PMC6942705

[59] Zhang G Kong X Wang M Zhao H Han S Hu R. Axl is a marker for epithelial-mesenchymal transition in esophageal squamous cell carcinoma. Oncol Lett (2018) 15(2):1900–6. doi: 10.3892/ol.2017.7443 PMC5774429 29434888

[60] Vandamme N Denecker G Bruneel K Blancke G Akay O Taminau J. The emt transcription factor zeb2 promotes proliferation of primary and metastatic melanoma while suppressing an invasive, mesenchymal-like phenotype. Cancer Res (2020) 80(14):2983–95. doi: 10.1158/0008-5472.CAN-19-2373 32503808

[61] Pedri D Karras P Landeloos E Marine JC Rambow F. Epithelial-to-mesenchymal-like transition events in melanoma. FEBS J (2022) 289(5):1352–68. doi: 10.1111/febs.16021 33999497

[62] Paluncic J Kovacevic Z Jansson PJ Kalinowski D Merlot AM Huang ML. Roads to melanoma: key pathways and emerging players in melanoma progression and oncogenic signaling. Biochim Biophys Acta (2016) 1863(4):770–84. doi: 10.1016/j.bbamcr.2016.01.025 26844774

[63] Wehbe M Soudja SM Mas A Chasson L Guinamard R de Tenbossche CP. Epithelial-mesenchymal-transition-like and tgfbeta pathways associated with autochthonous inflammatory melanoma development in mice. PloS One (2012) 7(11):e49419. doi: 10.1371/journal.pone.0049419 23173060 PMC3500287

[64] Hu X Yuan L Ma T. Mechanisms of jak-stat signaling pathway mediated by cxcl8 gene silencing on epithelial-mesenchymal transition of human cutaneous melanoma cells. Oncol Lett (2020) 20(2):1973–81. doi: 10.3892/ol.2020.11706 PMC7377181 32724443

[65] Wei CY Zhu MX Yang YW Zhang PF Yang X Peng R. Downregulation of rnf128 activates wnt/beta-catenin signaling to induce cellular emt and stemness *via* Cd44 and Cttn ubiquitination in melanoma. J Hematol Oncol (2019) 12(1):21. doi: 10.1186/s13045-019-0711-z 30832692 PMC6399928

[66] Bai X Fisher DE Flaherty KT. Cell-state dynamics and therapeutic resistance in melanoma from the perspective of mitf and ifngamma pathways. Nat Rev Clin Oncol (2019) 16(9):549–62. doi: 10.1038/s41571-019-0204-6 PMC7185899 30967646

[67] Yang Z Qi Y Lai N Zhang J Chen Z Liu M. Notch1 signaling in melanoma cells promoted tumor-induced immunosuppression *via* upregulation of tgf-beta1. J Exp Clin Cancer Res (2018) 37(1):1. doi: 10.1186/s13046-017-0664-4 29301578 PMC5755139

[68] Janghorban M Xin L Rosen JM Zhang XH. Notch signaling as a regulator of the tumor immune response: to target or not to target? Front Immunol (2018) 9:1649. doi: 10.3389/fimmu.2018.01649 30061899 PMC6055003

[69] Guri Y Nordmann TM Roszik J. Mtor at the transmitting and receiving ends in tumor immunity. Front Immunol (2018) 9:578. doi: 10.3389/fimmu.2018.00578 29662490 PMC5890199

[70] Caforio M de Billy E De Angelis B Iacovelli S Quintarelli C Paganelli V. Pi3k/akt pathway: the indestructible role of a vintage target as a support to the most recent immunotherapeutic approaches. Cancers (Basel) (2021) 13(16):4040. doi: 10.3390/cancers13164040 34439194 PMC8392360

[71] Bent EH Gilbert LA Hemann MT. A senescence secretory switch mediated by pi3k/akt/mtor activation controls chemoprotective endothelial secretory responses. Genes Dev (2016) 30(16):1811–21. doi: 10.1101/gad.284851.116 PMC5024680 27566778

[72] Tucci M Passarelli A Mannavola F Felici C Stucci LS Cives M. Immune system evasion as hallmark of melanoma progression: the role of dendritic cells. Front Oncol (2019) 9:1148. doi: 10.3389/fonc.2019.01148 31750245 PMC6848379

[73] Atefi M Avramis E Lassen A Wong DJ Robert L Foulad D. Effects of mapk and pi3k pathways on pd-L1 expression in melanoma. Clin Cancer Res (2014) 20(13):3446–57. doi: 10.1158/1078-0432.CCR-13-2797 PMC4079734 24812408

[74] Sumimoto H Imabayashi F Iwata T Kawakami Y. The braf-mapk signaling pathway is essential for cancer-immune evasion in human melanoma cells. J Exp Med (2006) 203(7):1651–6. doi: 10.1084/jem.20051848 PMC2118331 16801397

[75] Ott PA Bhardwaj N. Impact of mapk pathway activation in braf(V600) melanoma on T cell and dendritic cell function. Front Immunol (2013) 4:346. doi: 10.3389/fimmu.2013.00346 24194739 PMC3809567

[76] Mandala M De Logu F Merelli B Nassini R Massi D. Immunomodulating property of mapk inhibitors: from translational knowledge to clinical implementation. Lab Invest (2017) 97(2):166–75. doi: 10.1038/labinvest.2016.132 27991907

[77] Laine A Labiad O Hernandez-Vargas H This S Sanlaville A Leon S. Regulatory T cells promote cancer immune-escape through integrin alphavbeta8-mediated Tgf-beta activation. Nat Commun (2021) 12(1):6228. doi: 10.1038/s41467-021-26352-2 34711823 PMC8553942

[78] Diaz-Valdes N Basagoiti M Dotor J Aranda F Monreal I Riezu-Boj JI. Induction of monocyte chemoattractant protein-1 and interleukin-10 by tgfbeta1 in melanoma enhances tumor infiltration and immunosuppression. Cancer Res (2011) 71(3):812–21. doi: 10.1158/0008-5472.CAN-10-2698 21159663

[79] Perrot CY Javelaud D Mauviel A. Insights into the transforming growth factor-beta signaling pathway in cutaneous melanoma. Ann Dermatol (2013) 25(2):135–44. doi: 10.5021/ad.2013.25.2.135 PMC3662904 23717002

[80] Busse A Keilholz U. Role of tgf-beta in melanoma. Curr Pharm Biotechnol (2011) 12(12):2165–75. doi: 10.2174/138920111798808437 21619542

[81] Ma D Niederkorn JY. Transforming growth factor-beta down-regulates major histocompatibility complex class I antigen expression and increases the susceptibility of uveal melanoma cells to natural killer cell-mediated cytolysis. Immunology (1995) 86(2):263–9. PMC1384005 7490128

[82] Luond F Pirkl M Hisano M Prestigiacomo V Kalathur RK Beerenwinkel N. Hierarchy of tgfbeta/smad, hippo/yap/taz, and wnt/beta-catenin signaling in melanoma phenotype switching. Life Sci Alliance (2022) 5(2). doi: 10.26508/lsa.202101010 PMC8616544 34819356

[83] Li X Xiang Y Li F Yin C Li B Ke X. Wnt/beta-catenin signaling pathway regulating T cell-inflammation in the tumor microenvironment. Front Immunol (2019) 10:2293. doi: 10.3389/fimmu.2019.02293 31616443 PMC6775198

[84] Douglass SM Fane ME Sanseviero E Ecker BL Kugel CH 3rd Behera R. Myeloid-derived suppressor cells are a major source of Wnt5a in the melanoma microenvironment and depend on Wnt5a for full suppressive activity. Cancer Res (2021) 81(3):658–70. doi: 10.1158/0008-5472.CAN-20-1238 PMC8330365 33262126

[85] Ekstrom EJ Bergenfelz C von Bulow V Serifler F Carlemalm E Jonsson G. Wnt5a induces release of exosomes containing pro-angiogenic and immunosuppressive factors from Malignant melanoma cells. Mol Cancer (2014) 13:88. doi: 10.1186/1476-4598-13-88 24766647 PMC4022450

[86] Barbero G Castro MV Villanueva MB Quezada MJ Fernandez NB DeMorrow S. An autocrine wnt5a loop promotes nf-kappab pathway activation and cytokine/chemokine secretion in melanoma. Cells (2019) 8(9):1060. doi: 10.3390/cells8091060 31510045 PMC6770184

[87] Zhang CX Ye SB Ni JJ Cai TT Liu YN Huang DJ. Sting signaling remodels the tumor microenvironment by antagonizing myeloid-derived suppressor cell expansion. Cell Death Differ (2019) 26(11):2314–28. doi: 10.1038/s41418-019-0302-0 PMC6889506 30816302

[88] Wu R Wang C Li Z Xiao J Li C Wang X. Sox2 promotes resistance of melanoma with Pd-L1 high expression to T-cell-mediated cytotoxicity that can be reversed by Saha. J Immunother Cancer (2020) 8(2). doi: 10.1136/jitc-2020-001037 PMC7651737 33158915

[89] Ravindran Menon D Li Y Yamauchi T Osborne DG Vaddi PK Wempe MF. Egcg inhibits tumor growth in melanoma by targeting jak-stat signaling and its downstream Pd-L1/Pd-L2-Pd1 axis in tumors and enhancing cytotoxic T-cell responses. Pharm (Basel) (2021) 14(11):1081. doi: 10.3390/ph14111081 PMC8618268 34832863

[90] Gotthardt D Putz EM Straka E Kudweis P Biaggio M Poli V. Loss of stat3 in murine Nk cells enhances nk cell-dependent tumor surveillance. Blood (2014) 124(15):2370–9. doi: 10.1182/blood-2014-03-564450 25185262

[91] Frangieh CJ Melms JC Thakore PI Geiger-Schuller KR Ho P Luoma AM. Multimodal pooled perturb-cite-seq screens in patient models define mechanisms of cancer immune evasion. Nat Genet (2021) 53(3):332–41. doi: 10.1038/s41588-021-00779-1 PMC8376399 33649592

[92] Wellbrock C Weisser C Hassel JC Fischer P Becker J Vetter CS. Stat5 contributes to interferon resistance of melanoma cells. Curr Biol (2005) 15(18):1629–39. doi: 10.1016/j.cub.2005.08.036 16169484

[93] Kortylewski M Jove R Yu H. Targeting stat3 affects melanoma on multiple fronts. Cancer Metastasis Rev (2005) 24(2):315–27. doi: 10.1007/s10555-005-1580-1 15986140

[94] Kaler P Augenlicht L Klampfer L. Macrophage-derived il-1beta stimulates wnt signaling and growth of colon cancer cells: A crosstalk interrupted by vitamin D3. Oncogene (2009) 28(44):3892–902. doi: 10.1038/onc.2009.247 PMC2783659 19701245

[95] Ortenberg R Galore-Haskel G Greenberg I Zamlin B Sapoznik S Greenberg E. Ceacam1 promotes melanoma cell growth through Sox-2. Neoplasia (2014) 16(5):451–60. doi: 10.1016/j.neo.2014.05.003 PMC4198694 24931667

[96] Rosenbaum SR Tiago M Caksa S Capparelli C Purwin TJ Kumar G. Sox10 requirement for melanoma tumor growth is due, in part, to immune-mediated effects. Cell Rep (2021) 37(10):110085. doi: 10.1016/j.celrep.2021.110085 34879275 PMC8720266

[97] Malissen N Macagno N Granjeaud S Granier C Moutardier V Gaudy-Marqueste C. Hvem has a broader expression than pd-L1 and constitutes a negative prognostic marker and potential treatment target for melanoma. Oncoimmunology (2019) 8(12):e1665976. doi: 10.1080/2162402X.2019.1665976 31741766 PMC6844309

[98] Yokoyama S Takahashi A Kikuchi R Nishibu S Lo JA Hejna M. Sox10 regulates melanoma immunogenicity through an irf4-irf1 axis. Cancer Res (2021) 81(24):6131–41. doi: 10.1158/0008-5472.CAN-21-2078 PMC8678351 34728538

[99] Kudo-Saito C Shirako H Ohike M Tsukamoto N Kawakami Y. Ccl2 is critical for immunosuppression to promote cancer metastasis. Clin Exp Metastasis (2013) 30(4):393–405. doi: 10.1007/s10585-012-9545-6 23143679

[100] Kudo-Saito C Shirako H Takeuchi T Kawakami Y. Cancer metastasis is accelerated through immunosuppression during snail-induced emt of cancer cells. Cancer Cell (2009) 15(3):195–206. doi: 10.1016/j.ccr.2009.01.023 19249678

[101] Jinushi M Nakazaki Y Carrasco DR Draganov D Souders N Johnson M. Milk fat globule egf-8 promotes melanoma progression through coordinated akt and twist signaling in the tumor microenvironment. Cancer Res (2008) 68(21):8889–98. doi: 10.1158/0008-5472.CAN-08-2147 18974133

[102] Tittarelli A Navarrete M Lizana M Hofmann-Vega F Salazar-Onfray F. Hypoxic melanoma cells deliver micrornas to dendritic cells and cytotoxic T lymphocytes through connexin-43 channels. Int J Mol Sci (2020) 21(20):7567. doi: 10.3390/ijms21207567 33066331 PMC7589225

[103] Guo Y Lu X Chen Y Rendon B Mitchell RA Cuatrecasas M. Zeb1 induces immune checkpoints to form an immunosuppressive envelope around invading cancer cells. Sci Adv (2021) 7(21). doi: 10.1126/sciadv.abd7455 PMC8139582 34020945

[104] Chen L Gibbons DL Goswami S Cortez MA Ahn YH Byers LA. Metastasis is regulated via microrna-200/zeb1 axis control of tumour cell pd-L1 expression and intratumoral immunosuppression. Nat Commun (2014) 5:5241. doi: 10.1038/ncomms6241 25348003 PMC4212319

[105] Sanchez-Del-Campo L Marti-Diaz R Montenegro MF Gonzalez-Guerrero R Hernandez-Caselles T Martinez-Barba E. Mitf induces escape from innate immunity in melanoma. J Exp Clin Cancer Res (2021) 40(1):117. doi: 10.1186/s13046-021-01916-8 33789714 PMC8015040

[106] Mahmoud F Shields B Makhoul I Avaritt N Wong HK Hutchins LF. Immune surveillance in melanoma: from immune attack to melanoma escape and even counterattack. Cancer Biol Ther (2017) 18(7):451–69. doi: 10.1080/15384047.2017.1323596 PMC5639850 28513269

[107] Wiedemann GM Aithal C Kraechan A Heise C Cadilha BL Zhang J. Microphthalmia-associated transcription factor (Mitf) regulates immune cell migration into melanoma. Transl Oncol (2019) 12(2):350–60. doi: 10.1016/j.tranon.2018.10.014 PMC6290759 30502589

[108] Horn LA Fousek K Palena C. Tumor plasticity and resistance to immunotherapy. Trends Cancer (2020) 6(5):432–41. doi: 10.1016/j.trecan.2020.02.001 PMC7192950 32348738

[109] Muller J Krijgsman O Tsoi J Robert L Hugo W Song C. Low mitf/axl ratio predicts early resistance to multiple targeted drugs in melanoma. Nat Commun (2014) 5:5712. doi: 10.1038/ncomms6712 25502142 PMC4428333

[110] Lehmann J Caduff N Krzywinska E Stierli S Salas-Bastos A Loos B. Escape from nk cell tumor surveillance by ngfr-induced lipid remodeling in melanoma. Sci Adv (2023) 9(2):eadc8825. doi: 10.1126/sciadv.adc8825 36638181 PMC9839334

[111] Benboubker V Boivin F Dalle S Caramel J. Cancer cell phenotype plasticity as a driver of immune escape in melanoma. Front Immunol (2022) 13:873116. doi: 10.3389/fimmu.2022.873116 35432344 PMC9012258

[112] Huang F Santinon F Flores Gonzalez RE Del Rincon SV. Melanoma plasticity: promoter of metastasis and resistance to therapy. Front Oncol (2021) 11:756001. doi: 10.3389/fonc.2021.756001 34604096 PMC8481945

[113] Diener J Sommer L. Reemergence of neural crest stem cell-like states in melanoma during disease progression and treatment. Stem Cells Transl Med (2021) 10(4):522–33. doi: 10.1002/sctm.20-0351 PMC7980219 33258291

[114] Kulesa PM Morrison JA Bailey CM. The neural crest and cancer: A developmental spin on melanoma. Cells Tissues Organs (2013) 198(1):12–21. doi: 10.1159/000348418 23774755 PMC3809092

[115] Bedogni B. Notch signaling in melanoma: interacting pathways and stromal influences that enhance notch targeting. Pigment Cell Melanoma Res (2014) 27(2):162–8. doi: 10.1111/pcmr.12194 24330305

[116] Pearlman RL Montes de Oca MK Pal HC Afaq F. Potential therapeutic targets of epithelial-mesenchymal transition in melanoma. Cancer Lett (2017) 391:125–40. doi: 10.1016/j.canlet.2017.01.029 PMC5371401 28131904

[117] Tudrej KB Czepielewska E Kozlowska-Wojciechowska M. Sox10-mitf pathway activity in melanoma cells. Arch Med Sci (2017) 13(6):1493–503. doi: 10.5114/aoms.2016.60655 PMC5701683 29181082

[118] Heppt MV Wang JX Hristova DM Wei Z Li L Evans B. Msx1-induced neural crest-like reprogramming promotes melanoma progression. J Invest Dermatol (2018) 138(1):141–9. doi: 10.1016/j.jid.2017.05.038 PMC5902795 28927893

[119] Larribere L Utikal J. Stem cell-derived models of neural crest are essential to understand melanoma progression and therapy resistance. Front Mol Neurosci (2019) 12:111. doi: 10.3389/fnmol.2019.00111 31118886 PMC6506783

[120] Wessely A Steeb T Berking C Heppt MV. How neural crest transcription factors contribute to melanoma heterogeneity, cellular plasticity, and treatment resistance. Int J Mol Sci (2021) 22(11):5761. doi: 10.3390/ijms22115761 34071193 PMC8198848

[121] Turco MY Furia L Dietze A Fernandez Diaz L Ronzoni S Sciullo A. Cellular heterogeneity during embryonic stem cell differentiation to epiblast stem cells is revealed by the shcd/ralp adaptor protein. Stem Cells (2012) 30(11):2423–36. doi: 10.1002/stem.1217 PMC3533801 22948967

[122] Heppt MV Wessely A Hornig E Kammerbauer C Graf SA Besch R. Hdac2 is involved in the regulation of brn3a in melanocytes and melanoma. Int J Mol Sci (2022) 23(2):849. doi: 10.3390/ijms23020849 35055045 PMC8778714

[123] Hohenauer T Berking C Schmidt A Haferkamp S Senft D Kammerbauer C. The neural crest transcription factor Brn3a is expressed in melanoma and required for cell cycle progression and survival. EMBO Mol Med (2013) 5(6):919–34. doi: 10.1002/emmm.201201862 PMC3779452 23666755

[124] Deng Z Wang H Liu J Deng Y Zhang N. Comprehensive understanding of anchorage-independent survival and its implication in cancer metastasis. Cell Death Dis (2021) 12(7):629. doi: 10.1038/s41419-021-03890-7 34145217 PMC8213763

[125] Fazio M van Rooijen E Dang M van de Hoek G Ablain J Mito JK. Satb2 induction of a neural crest mesenchyme-like program drives melanoma invasion and drug resistance. Elife (2021) 10. doi: 10.7554/eLife.64370 PMC7880683 33527896

[126] Lee JH Shklovskaya E Lim SY Carlino MS Menzies AM Stewart A. Transcriptional downregulation of mhc class I and melanoma de- differentiation in resistance to pd-1 inhibition. Nat Commun (2020) 11(1):1897. doi: 10.1038/s41467-020-15726-7 32312968 PMC7171183

[127] Liu J Rebecca VW Kossenkov AV Connelly T Liu Q Gutierrez A. Neural crest-like stem cell transcriptome analysis identifies lpar1 in melanoma progression and therapy resistance. Cancer Res (2021) 81(20):5230–41. doi: 10.1158/0008-5472.CAN-20-1496 PMC8530965 34462276

[128] Rasheed SAK Subramanyan LV Lim WK Udayappan UK Wang M Casey PJ. The emerging roles of galpha12/13 proteins on the hallmarks of cancer in solid tumors. Oncogene (2022) 41(2):147–58. doi: 10.1038/s41388-021-02069-w PMC8732267 34689178

[129] Kim MH Kim CG Kim SK Shin SJ Choe EA Park SH. Yap-induced pd-L1 expression drives immune evasion in Brafi-resistant melanoma. Cancer Immunol Res (2018) 6(3):255–66. doi: 10.1158/2326-6066.CIR-17-0320 29382670

[130] Li J Yang R Yang H Chen S Wang L Li M. Ncam regulates the proliferation, apoptosis, autophagy, emt, and migration of human melanoma cells *via* the Src/Akt/Mtor/Cofilin signaling pathway. J Cell Biochem (2020) 121(2):1192–204. doi: 10.1002/jcb.29353 31468584

[131] Shen C He Y Chen Q Feng H Williams TM Lu Y. Narrative review of emerging roles for Akt-Mtor signaling in cancer radioimmunotherapy. Ann Transl Med (2021) 9(20):1596. doi: 10.21037/atm-21-4544 34790802 PMC8576660

[132] Schille C Bayerlova M Bleckmann A Schambony A. Ror2 signaling is required for local upregulation of Gdf6 and activation of bmp signaling at the neural plate border. Development (2016) 143(17):3182–94. doi: 10.1242/dev.135426 27578181

[133] Venkatesan AM Vyas R Gramann AK Dresser K Gujja S Bhatnagar S. Ligand-activated bmp signaling inhibits cell differentiation and death to promote melanoma. J Clin Invest (2018) 128(1):294–308. doi: 10.1172/JCI92513 29202482 PMC5749509

[134] Gramann AK Frantz WT Dresser K Gomes CBF Lian CG Deng A. Bmp signaling promotes neural crest identity and accelerates melanoma onset. J Invest Dermatol (2021) 141(8):2067–70 e1. doi: 10.1016/j.jid.2021.01.021 33610560

[135] Rambow F Rogiers A Marin-Bejar O Aibar S Femel J Dewaele M. Toward minimal residual disease-directed therapy in melanoma. Cell (2018) 174(4):843–55 e19. doi: 10.1016/j.cell.2018.06.025 30017245

[136] Chou B Hiromatsu K Okano S Ishii K Duan X Sakai T. Antiangiogenic tumor therapy by DNA vaccine inducing aquaporin-1-specific ctl based on ubiquitin-proteasome system in mice. J Immunol (2012) 189(4):1618–26. doi: 10.4049/jimmunol.1101971 22802414

[137] Furuta J Inozume T Harada K Shimada S. Cd271 on melanoma cell is an ifn-gamma-inducible immunosuppressive factor that mediates downregulation of melanoma antigens. J Invest Dermatol (2014) 134(5):1369–77. doi: 10.1038/jid.2013.490 24226422

[138] Hu J Verkman AS. Increased migration and metastatic potential of tumor cells expressing aquaporin water channels. FASEB J (2006) 20(11):1892–4. doi: 10.1096/fj.06-5930fje 16818469

[139] Andreucci E Peppicelli S Ruzzolini J Bianchini F Biagioni A Papucci L. The acidic tumor microenvironment drives a stem-like phenotype in melanoma cells. J Mol Med (Berl) (2020) 98(10):1431–46. doi: 10.1007/s00109-020-01959-y PMC7525286 32803272

[140] Pastorekova S Gillies RJ. The role of carbonic anhydrase ix in cancer development: links to hypoxia, acidosis, and beyond. Cancer Metastasis Rev (2019) 38(1-2):65–77. doi: 10.1007/s10555-019-09799-0 31076951 PMC6647366

[141] Peppicelli S Ruzzolini J Bianchini F Andreucci E Nediani C Laurenzana A. Anoikis resistance as a further trait of acidic-adapted melanoma cells. J Oncol (2019) 2019:8340926. doi: 10.1155/2019/8340926 31275384 PMC6582804

[142] Boussadia Z Lamberti J Mattei F Pizzi E Puglisi R Zanetti C. Acidic microenvironment plays a key role in human melanoma progression through a sustained exosome mediated transfer of clinically relevant metastatic molecules. J Exp Clin Cancer Res (2018) 37(1):245. doi: 10.1186/s13046-018-0915-z 30290833 PMC6173926

[143] Huber V Camisaschi C Berzi A Ferro S Lugini L Triulzi T. Cancer acidity: an ultimate frontier of tumor immune escape and a novel target of immunomodulation. Semin Cancer Biol (2017) 43:74–89. doi: 10.1016/j.semcancer.2017.03.001 28267587

[144] Scharping NE Rivadeneira DB Menk AV Vignali PDA Ford BR Rittenhouse NL. Mitochondrial stress induced by continuous stimulation under hypoxia rapidly drives T cell exhaustion. Nat Immunol (2021) 22(2):205–15. doi: 10.1038/s41590-020-00834-9 PMC7971090 33398183

[145] Wagner M Ealey KN Tetsu H Kiniwa T Motomura Y Moro K. Tumor-derived lactic acid contributes to the paucity of intratumoral ilc2s. Cell Rep (2020) 30(8):2743–57 e5. doi: 10.1016/j.celrep.2020.01.103 32101749

[146] Harel M Ortenberg R Varanasi SK Mangalhara KC Mardamshina M Markovits E. Proteomics of melanoma response to immunotherapy reveals mitochondrial dependence. Cell (2019) 179(1):236–50 e18. doi: 10.1016/j.cell.2019.08.012 31495571 PMC7993352

[147] Fuhrmann DC Olesch C Kurrle N Schnutgen F Zukunft S Fleming I. Chronic hypoxia enhances beta-oxidation-dependent electron transport *via* electron transferring flavoproteins. Cells (2019) 8(2):172. doi: 10.3390/cells8020172 30781698 PMC6406996

[148] Munir R Lisec J Swinnen JV Zaidi N. Lipid metabolism in cancer cells under metabolic stress. Br J Cancer (2019) 120(12):1090–8. doi: 10.1038/s41416-019-0451-4 PMC6738079 31092908

[149] Filipp FV Scott DA Ronai ZA Osterman AL Smith JW. Reverse tca cycle flux through isocitrate dehydrogenases 1 and 2 is required for lipogenesis in hypoxic melanoma cells. Pigment Cell Melanoma Res (2012) 25(3):375–83. doi: 10.1111/j.1755-148X.2012.00989.x PMC3329592 22360810

[150] Liu Z Liu W Wang W Ma Y Wang Y Drum DL. Cpt1a-mediated fatty acid oxidation confers cancer cell resistance to immune-mediated cytolytic killing. Proc Natl Acad Sci U.S.A. (2023) 120(39):e2302878120. doi: 10.1073/pnas.2302878120 37722058 PMC10523454

[151] Yang NY Pasquale EB Owen LB Ethell IM. The ephb4 receptor-tyrosine kinase promotes the migration of melanoma cells through rho-mediated actin cytoskeleton reorganization. J Biol Chem (2006) 281(43):32574–86. doi: 10.1074/jbc.M604338200 16950769

[152] Neuber C Troster A Loser R Belter B Schwalbe H Pietzsch J. The pyrazolo[3,4-D]Pyrimidine-based kinase inhibitor nvp-bhg712: effects of regioisomers on tumor growth, perfusion, and hypoxia in ephb4-positive A375 melanoma xenografts. Molecules (2020) 25(21):5115. doi: 10.3390/molecules25215115 33153234 PMC7662635

[153] Filimon A Preda IA Boloca AF Negroiu G. Interleukin-8 in melanoma pathogenesis, prognosis and therapy-an integrated view into other neoplasms and chemokine networks. Cells (2021) 11(1):120. doi: 10.3390/cells11010120 35011682 PMC8750532

[154] Corbet C Bastien E Santiago de Jesus JP Dierge E Martherus R Vander Linden C. Tgfbeta2-induced formation of lipid droplets supports acidosis-driven emt and the metastatic spreading of cancer cells. Nat Commun (2020) 11(1):454. doi: 10.1038/s41467-019-14262-3 31974393 PMC6978517

[155] Bhatia S Oweida A Lennon S Darragh LB Milner D Phan AV. Inhibition of ephb4-ephrin-B2 signaling reprograms the tumor immune microenvironment in head and neck cancers. Cancer Res (2019) 79(10):2722–35. doi: 10.1158/0008-5472.CAN-18-3257 PMC6522285 30894369

[156] Liu C He S Zhang J Li S Chen J Han C. Silencing tcf4 sensitizes melanoma cells to vemurafenib through inhibiting glut3-mediated glycolysis. Onco Targets Ther (2020) 13:4905–15. doi: 10.2147/OTT.S245531 PMC7269014 32581551

[157] Pozniak J Pedri D Landeloos E Herck YV Antoranz A Karras P. A TCF4-dependent gene regulatory network confers resistance to immunotherapy in melanoma. Cell. (2024) 187(1):166–83.e25. doi: 10.1016/j.cell.2023.11.037 38181739

[158] Pinheiro C Miranda-Goncalves V Longatto-Filho A Vicente AL Berardinelli GN Scapulatempo-Neto C. The metabolic microenvironment of melanomas: prognostic value of mct1 and mct4. Cell Cycle (2016) 15(11):1462–70. doi: 10.1080/15384101.2016.1175258 PMC4934068 27105345

[159] Tasdogan A Faubert B Ramesh V Ubellacker JM Shen B Solmonson A. Metabolic heterogeneity confers differences in melanoma metastatic potential. Nature (2020) 577(7788):115–20. doi: 10.1038/s41586-019-1847-2 PMC6930341 31853067

[160] Malekan M Ebrahimzadeh MA Sheida F. The role of hypoxia-inducible factor-1alpha and its signaling in melanoma. BioMed Pharmacother (2021) 141:111873. doi: 10.1016/j.biopha.2021.111873 34225012

[161] Janji B Chouaib S. “Suffocating” Tumors by blocking adaptation to hypoxia: A new headway in melanoma immunotherapy. Oncoimmunology (2021) 10(1):1968611. doi: 10.1080/2162402X.2021.1968611 34527430 PMC8437449

[162] You L Wu W Wang X Fang L Adam V Nepovimova E. The role of hypoxia-inducible factor 1 in tumor immune evasion. Med Res Rev (2021) 41(3):1622–43. doi: 10.1002/med.21771 33305856

[163] Brozyna AA Jozwicki W Jetten AM Slominski AT. On the relationship between vdr, roralpha and rorgamma receptors expression and hif1-alpha levels in human melanomas. Exp Dermatol (2019) 28(9):1036–43. doi: 10.1111/exd.14002 PMC6715521 31287590

[164] Chen C Gao FH. Th17 cells paradoxical roles in melanoma and potential application in immunotherapy. Front Immunol (2019) 10:187. doi: 10.3389/fimmu.2019.00187 30800130 PMC6375889

[165] Xu Q Briggs J Park S Niu G Kortylewski M Zhang S. Targeting stat3 blocks both hif-1 and vegf expression induced by multiple oncogenic growth signaling pathways. Oncogene (2005) 24(36):5552–60. doi: 10.1038/sj.onc.1208719 16007214

[166] Shehade H Acolty V Moser M Oldenhove G. Cutting edge: hypoxia-inducible factor 1 negatively regulates th1 function. J Immunol (2015) 195(4):1372–6. doi: 10.4049/jimmunol.1402552 26179900

[167] Wei P Kou W Riaz F Zhang K Fu J Pan F. Combination therapy of hif1alpha inhibitors and treg depletion strengthen the anti-tumor immunity in mice. Eur J Immunol (2023) 53:e2250182. doi: 10.1002/eji.202250182 37615189

[168] Lequeux A Noman MZ Xiao M Van Moer K Hasmim M Benoit A. Targeting hif-1 alpha transcriptional activity drives cytotoxic immune effector cells into melanoma and improves combination immunotherapy. Oncogene (2021) 40(28):4725–35. doi: 10.1038/s41388-021-01846-x PMC8282500 34155342

[169] Kim NH Jeong JH Park YJ Shin HY Choi WK Lee K. Anti-tumor effect of idf-11774, an inhibitor of hypoxia-inducible factor-1, on melanoma. Biomol Ther (Seoul) (2022) 30(5):465–72. doi: 10.4062/biomolther.2022.061 PMC9424330 35712870

[170] Li C Wang Q Shen S Wei X Li G. Hif-1alpha/vegf signaling-mediated epithelial-mesenchymal transition and angiogenesis is critically involved in anti-metastasis effect of luteolin in melanoma cells. Phytother Res (2019) 33(3):798–807. doi: 10.1002/ptr.6273 30653763 PMC6590488

[171] Wang J Ning D Xie D Chen X Cao X Wan C. Functional involvement of adra1d in cutaneous melanoma progression and angiogenesis. Cell Mol Biol (Noisy-le-grand) (2023) 69(5):44–50. doi: 10.14715/cmb/2023.69.5.8 37571902

[172] Zheng Y Chen H Zhao Y Zhang X Liu J Pan Y. Knockdown of fbxo22 inhibits melanoma cell migration, invasion and angiogenesis *via* the hif-1alpha/vegf pathway. Invest New Drugs (2020) 38(1):20–8. doi: 10.1007/s10637-019-00761-z 30887251

[173] Yang H Grossniklaus HE Yan Y Offermann M Van Meir E. Hif-inhibitor 64b induces necrosis and suppresses proliferation, metastasis and yap1 expression in mouse model of uveal melanoma. Invest Ophthalmol Visual Sci (2022) 63(7):2362–A0031-2362–A0031.

[174] Roy S Rizvi ZA Clarke AJ Macdonald F Pandey A Zaiss DMW. Egfr-hif1alpha signaling positively regulates the differentiation of il-9 producing T helper cells. Nat Commun (2021) 12(1):3182. doi: 10.1038/s41467-021-23042-x 34075041 PMC8169867

[175] de Almeida PE Mak J Hernandez G Jesudason R Herault A Javinal V. Anti-vegf treatment enhances cd8(+) T-cell antitumor activity by amplifying hypoxia. Cancer Immunol Res (2020) 8(6):806–18. doi: 10.1158/2326-6066.CIR-19-0360 32238381

[176] Lim SA Moon Y Shin MH Kim TJ Chae S Yee C. Hypoxia-driven hif-1alpha activation reprograms pre-activated nk cells towards highly potent effector phenotypes *via* erk/stat3 pathways. Cancers (Basel) (2021) 13(8):1904. doi: 10.3390/cancers13081904 33920906 PMC8071270

[177] Louphrasitthiphol P Ledaki I Chauhan J Falletta P Siddaway R Buffa FM. Mitf controls the tca cycle to modulate the melanoma hypoxia response. Pigment Cell Melanoma Res (2019) 32(6):792–808. doi: 10.1111/pcmr.12802 31207090 PMC6777998

[178] Bohme I Bosserhoff A. Extracellular acidosis triggers a senescence-like phenotype in human melanoma cells. Pigment Cell Melanoma Res (2020) 33(1):41–51. doi: 10.1111/pcmr.12811 31310445

[179] Cao L Li W Yang X Zhang W Li M Zhang H. Inhibition of host ogr1 enhances effector cd8(+) T-cell function by modulating acidic microenvironment. Cancer Gene Ther (2021) 28(10-11):1213–24. doi: 10.1038/s41417-021-00354-0 PMC8571096 34158625

[180] Damaghi M Wojtkowiak JW Gillies RJ. Ph sensing and regulation in cancer. Front Physiol (2013) 4:370. doi: 10.3389/fphys.2013.00370 24381558 PMC3865727

[181] Mori D Tsujikawa T Sugiyama Y Kotani SI Fuse S Ohmura G. Extracellular acidity in tumor tissue upregulates programmed cell death protein 1 expression on tumor cells *via* proton-sensing G protein-coupled receptors. Int J Cancer (2021) 149(12):2116–24. doi: 10.1002/ijc.33786 34460096

[182] Justus CR Yang LV. Gpr4 decreases B16f10 melanoma cell spreading and regulates focal adhesion dynamics through the G13/Rho signaling pathway. Exp Cell Res (2015) 334(1):100–13. doi: 10.1016/j.yexcr.2015.03.022 25845498

[183] Wilhelm I Fazakas C Molnar J Hasko J Vegh AG Cervenak L. Role of rho/rock signaling in the interaction of melanoma cells with the blood-brain barrier. Pigment Cell Melanoma Res (2014) 27(1):113–23. doi: 10.1111/pcmr.12169 24148763

[184] Limia CM Sauzay C Urra H Hetz C Chevet E Avril T. Emerging roles of the endoplasmic reticulum associated unfolded protein response in cancer cell migration and invasion. Cancers (Basel) (2019) 11(5):631. doi: 10.3390/cancers11050631 31064137 PMC6562633

[185] Kwon YJ Seo EB Kwon SH Lee SH Kim SK Park SK. Extracellular acidosis promotes metastatic potency via decrease of the bmal1 circadian clock gene in breast cancer. Cells (2020) 9(4):989. doi: 10.3390/cells9040989 32316196 PMC7226966

[186] Alexander RK Liou YH Knudsen NH Starost KA Xu C Hyde AL. Bmal1 integrates mitochondrial metabolism and macrophage activation. Elife (2020) 9. doi: 10.7554/eLife.54090 PMC7259948 32396064

[187] Tamaru T Takamatsu K. Circadian modification network of a core clock driver bmal1 to harmonize physiology from brain to peripheral tissues. Neurochem Int (2018) 119:11–6. doi: 10.1016/j.neuint.2017.12.013 29305918

[188] Li W Zhou Y. Lrig1 acts as a critical regulator of melanoma cell invasion, migration, and vasculogenic mimicry upon hypoxia by regulating Egfr/Erk-triggered epithelial-mesenchymal transition. Biosci Rep (2019) 39(1). doi: 10.1042/BSR20181165 PMC6328857 30487162

[189] Oberoi-Khanuja TK Karreman C Larisch S Rapp UR Rajalingam K. Role of melanoma inhibitor of apoptosis (Ml-iap) protein, a member of the baculoviral iap repeat (Bir) domain family, in the regulation of C-raf kinase and cell migration. J Biol Chem (2012) 287(34):28445–55. doi: 10.1074/jbc.M112.341297 PMC3436542 22711539

[190] Samanta D Huang TY Shah R Yang Y Pan F Semenza GL. Birc2 expression impairs anti-cancer immunity and immunotherapy efficacy. Cell Rep (2020) 32(8):108073. doi: 10.1016/j.celrep.2020.108073 32846130

[191] Vasilikos L Hanggi K Spilgies LM Kisele S Rufli S Wong WW. Loss of ciap1 in endothelial cells limits metastatic extravasation through tumor-derived lymphotoxin alpha. Cancers (Basel) (2021) 13(4):599. doi: 10.3390/cancers13040599 33546280 PMC7913358

[192] Varfolomeev E Blankenship JW Wayson SM Fedorova AV Kayagaki N Garg P. Iap antagonists induce autoubiquitination of C-iaps, Nf-Kappab activation, and Tnfalpha-dependent apoptosis. Cell (2007) 131(4):669–81. doi: 10.1016/j.cell.2007.10.030 18022362

[193] Touil Y Segard P Ostyn P Begard S Aspord C El Machhour R. Melanoma dormancy in a mouse model is linked to Gilz/Foxo3a-dependent quiescence of disseminated stem-like cells. Sci Rep (2016) 6:30405. doi: 10.1038/srep30405 27465291 PMC4964333

[194] Dong Z Yang J Li L Tan L Shi P Zhang J. Foxo3asirt6 axis suppresses aerobic glycolysis in melanoma. Int J Oncol (2020) 56(3):728–42. doi: 10.3892/ijo.2020.4964 PMC7010217 32124950

[195] Garcia-Peterson LM Ndiaye MA Chhabra G Singh CK Guzman-Perez G Iczkowski KA. Crispr/Cas9-mediated knockout of Sirt6 imparts remarkable antiproliferative response in human melanoma cells in vitro and in vivo. Photochem Photobiol (2020) 96(6):1314–20. doi: 10.1111/php.13305 PMC8262248 32621766

[196] Wang L Guo W Ma J Dai W Liu L Guo S. Aberrant Sirt6 expression contributes to melanoma growth: role of the autophagy paradox and Igf-Akt signaling. Autophagy (2018) 14(3):518–33. doi: 10.1080/15548627.2017.1384886 PMC5915046 29215322

[197] Jia Q Yang F Huang W Zhang Y Bao B Li K. Low levels of Sox2 are required for melanoma tumor-repopulating cell dormancy. Theranostics (2019) 9(2):424–35. doi: 10.7150/thno.29698 PMC6376184 30809284

[198] La T Chen S Guo T Zhao XH Teng L Li D. Visualization of endogenous P27 and Ki67 reveals the importance of a C-myc-driven metabolic switch in promoting survival of quiescent cancer cells. Theranostics (2021) 11(19):9605–22. doi: 10.7150/thno.63763 PMC8490506 34646389

[199] Touil Y Zuliani T Wolowczuk I Kuranda K Prochazkova J Andrieux J. The Pi3k/Akt signaling pathway controls the quiescence of the low-rhodamine123-retention cell compartment enriched for melanoma stem cell activity. Stem Cells (2013) 31(4):641–51. doi: 10.1002/stem.1333 23355370

[200] Kfoury A Armaro M Collodet C Sordet-Dessimoz J Giner MP Christen S. Ampk promotes survival of C-myc-positive melanoma cells by suppressing oxidative stress. EMBO J (2018) 37(5). doi: 10.15252/embj.201797673 PMC5830923 29440228

[201] Towler MC Hardie DG. Amp-activated protein kinase in metabolic control and insulin signaling. Circ Res (2007) 100(3):328–41. doi: 10.1161/01.RES.0000256090.42690.05 17307971

[202] Herzig S Shaw RJ. Ampk: guardian of metabolism and mitochondrial homeostasis. Nat Rev Mol Cell Biol (2018) 19(2):121–35. doi: 10.1038/nrm.2017.95 PMC5780224 28974774

[203] Andreucci E Pietrobono S Peppicelli S Ruzzolini J Bianchini F Biagioni A. Sox2 as a novel contributor of oxidative metabolism in melanoma cells. Cell Commun Signal (2018) 16(1):87. doi: 10.1186/s12964-018-0297-z 30466459 PMC6249961

[204] Girouard SD Laga AC Mihm MC Scolyer RA Thompson JF Zhan Q. Sox2 contributes to melanoma cell invasion. Lab Invest (2012) 92(3):362–70. doi: 10.1038/labinvest.2011.188 PMC3887365 22184093

[205] Mandalos N Rhinn M Granchi Z Karampelas I Mitsiadis T Economides AN. Sox2 acts as a rheostat of epithelial to mesenchymal transition during neural crest development. Front Physiol (2014) 5:345. doi: 10.3389/fphys.2014.00345 25309446 PMC4162359

[206] He F Wu H Zhou L Lin Q Cheng Y Sun YE. Tet2-mediated epigenetic drive for astrocyte differentiation from embryonic neural stem cells. Cell Death Discovery (2020) 6:30. doi: 10.1038/s41420-020-0264-5 32377393 PMC7190615

[207] Pan W Zhu S Qu K Meeth K Cheng J He K. The DNA methylcytosine dioxygenase tet2 sustains immunosuppressive function of tumor-infiltrating myeloid cells to promote melanoma progression. Immunity (2017) 47(2):284–97 e5. doi: 10.1016/j.immuni.2017.07.020 28813659 PMC5710009

[208] Bonvin E Radaelli E Bizet M Luciani F Calonne E Putmans P. Tet2-dependent hydroxymethylome plasticity reduces melanoma initiation and progression. Cancer Res (2019) 79(3):482–94. doi: 10.1158/0008-5472.CAN-18-1214 PMC6612266 30538121

[209] Morrison JA McLennan R Wolfe LA Gogol MM Meier S McKinney MC. Single-cell transcriptome analysis of avian neural crest migration reveals signatures of invasion and molecular transitions. Elife (2017) 6. doi: 10.7554/eLife.28415 PMC5728719 29199959

[210] Liu Y Lv J Liu J Liang X Jin X Xie J. Stat3/P53 pathway activation disrupts ifn-beta-induced dormancy in tumor-repopulating cells. J Clin Invest (2018) 128(3):1057–73. doi: 10.1172/JCI96329 PMC5824876 29431732

[211] Nichane M Ren X Bellefroid EJ. Self-regulation of stat3 activity coordinates cell-cycle progression and neural crest specification. EMBO J (2010) 29(1):55–67. doi: 10.1038/emboj.2009.313 19851287 PMC2808363

[212] Rapanotti MC Campione E Suarez Viguria TM Spallone G Costanza G Rossi P. Stem-mesenchymal signature cell genes detected in heterogeneous circulating melanoma cells correlate with disease stage in melanoma patients. Front Mol Biosci (2020) 7:92. doi: 10.3389/fmolb.2020.00092 32548126 PMC7272706

[213] Ng MF Simmons JL Boyle GM. Heterogeneity in melanoma. Cancers (Basel) (2022) 14(12):3030. doi: 10.3390/cancers14123030 35740696 PMC9221188

[214] Radke J Schumann E Onken J Koll R Acker G Bodnar B. Decoding molecular programs in melanoma brain metastases. Nat Commun (2022) 13(1):7304. doi: 10.1038/s41467-022-34899-x 36435874 PMC9701224

[215] Quail DF Joyce JA. The microenvironmental landscape of brain tumors. Cancer Cell (2017) 31(3):326–41. doi: 10.1016/j.ccell.2017.02.009 PMC5424263 28292436

[216] Pozzi S Scomparin A Ben-Shushan D Yeini E Ofek P Nahmad AD. Mcp-1/ccr2 axis inhibition sensitizes the brain microenvironment against melanoma brain metastasis progression. JCI Insight (2022) 7(17). doi: 10.1172/jci.insight.154804 PMC9536270 35980743

[217] Izraely S Ben-Menachem S Malka S Sagi-Assif O Bustos MA Adir O. The vicious cycle of melanoma-microglia crosstalk: inter-melanoma variations in the brain-metastasis-promoting Il-6/Jak/Stat3 signaling pathway. Cells (2023) 12(11):1513. doi: 10.3390/cells12111513 37296634 PMC10253015

[218] Biermann J Melms JC Amin AD Wang Y Caprio LA Karz A. Dissecting the treatment-naive ecosystem of human melanoma brain metastasis. Cell (2022) 185(14):2591–608 e30. doi: 10.1016/j.cell.2022.06.007 35803246 PMC9677434

[219] Fischer GM Jalali A Kircher DA Lee WC McQuade JL Haydu LE. Molecular profiling reveals unique immune and metabolic features of melanoma brain metastases. Cancer Discovery (2019) 9(5):628–45. doi: 10.1158/2159-8290.CD-18-1489 PMC6497554 30787016

[220] In GK Ribeiro JR Yin J Xiu J Bustos MA Ito F. Multi-omic profiling reveals discrepant immunogenic properties and a unique tumor microenvironment among melanoma brain metastases. NPJ Precis Oncol (2023) 7(1):120. doi: 10.1038/s41698-023-00471-z 37964004 PMC10646102

